# Evaluation of a reference antibody panel for prediction of cytokine release in humanised mouse models *in vivo*

**DOI:** 10.3389/fimmu.2026.1736130

**Published:** 2026-03-20

**Authors:** Deepa Rajagopal, Ka Seng Ieong, Ryan Mate, Sandrine Vessillier

**Affiliations:** 1 Immunotherapy, Biotherapeutics and Advanced Therapies Division, Science and Research, Medicines and Healthcare Products Regulatory Agency (MHRA), South Mimms, Hertfordshire, United Kingdom; 2 Analytical and Biological Sciences, Science and Research, Medicines, and Healthcare Products Regulatory Agency (MHRA), South Mimms, Hertfordshire, United Kingdom

**Keywords:** cytokine release, hazard identification, human peripheral blood mononuclear cells (PBMC), humanised mouse models, NOD-scid-gamma (NSG) mice, preclinical safety assessment, recombinant monoclonal antibodies, reference antibody panel

## Abstract

**Introduction:**

A diverse range of innovative biological therapies is being developed to treat various human diseases. The safety assessment of these biologics is a critical factor determining clinical success. Enhanced humanised mouse models have the potential to revolutionise immunotoxicological profiling by refining procedures for effective *in vivo* safety assessments.

**Methods:**

We evaluated a novel reference panel of recombinant monoclonal antibodies (mAbs), 19/156, with varying cytokine release (CR) potential to assess the sensitivity of specific humanised mouse models. The *in vitro* CR capacity of the reference panel has previously been evaluated in an international collaborative study. We present here the *in vivo* assessment of the reference Ab panel using NOD-scid-gamma (NSG) mice reconstituted with either umbilical cord-derived hematopoietic human (CD34^+^) stem cells (HSC) or human peripheral blood mononuclear cells (PBMC) from healthy donors.

**Results:**

Our manuscript discusses a comparative evaluation of both forms of engraftment and the CR patterns in response to the reference panel. The *in vivo* CR pattern is discussed in relation to *in vitro* assays using the same PBMC donor cohort. The manuscript discusses the utility of these humanised mice as a model for translational use in hazard identification and preclinical safety assessment.

**Discussion:**

The results highlight the importance of incorporating standardised reference materials to evaluate, qualify, and harmonise preclinical models for translational use. This approach aims to enhance the predictability and reliability of both *in vitro* and *in vivo* safety assessments, thereby supporting the development of safe and effective biological therapies.

## 1 Introduction

Advanced biological therapies, including multi-functional or multi-specific antibodies (Abs) and cellular chimeric antigen receptor (CAR) T-cell therapies, have complex mechanisms of action that may lead to immune-mediated or immune-related side effects (1–3). These therapies can exert their immunomodulatory effects by either enhancing or suppressing immune responses. In the context of cancer immunotherapy, specific approaches are employed to promote T-cell activation and/or reinvigoration, for an effective anti-tumour response generation. The field is progressing rapidly, with advancements in agonistic and blocking monoclonal Abs (mAbs) targeting T-cell costimulatory receptors, as well as the development of bi-specifics, cell engagers, and T cell-based therapies. However, achieving efficacy by pushing the limits of the immune system by releasing ‘brakes’ on T-cell activation also presents significant safety concerns as was evidenced with the TGN1412, CD28- super-agonist (SA) disaster in 2006. An uncontrolled expansion of T-cells leading to proinflammatory cytokine release and T-cell tissue infiltration caused a life threatening systemic inflammatory response termed as cytokine release (CR) syndrome (CRS) in clinical trial patients (4, 5). CRS initially triggered by the massive release of cytokines from targeted and bystander immune cells, could lead to multiorgan failure and death in extreme cases (6, 7). The development of predictive models for potential adverse events is therefore essential during preclinical research. Traditional animal models are valuable for clinical translation; however species-specific molecular and immunological differences could often constrain their utility in hazard identification. Advanced higher animal model on the other hand may often fail to capture the entire spectrum of toxicities related to immune activation as shown when preclinical safety testing for TGN1412 in macaques failed to predict the adverse response observed in clinical trials. Therefore, the development of predictive assays applicable to a wide spectrum of biotherapeutics, integrating the *in vivo* complexities of immune pathways is crucial for harmonising immunotoxicological assessment of novel biotherapeutics. Improved humanised mouse models have the potential to enhance immunotoxicological profiling, offering an alternative to non-human primate (NHP) and guinea pig animal models, potentially contributing to reduction and refinement in preclinical safety evaluations. These models could bridge the differences between mice and humans and be a useful system for evaluation of human-specific immune responses during therapeutic development. Understanding and predicting toxicities early in the drug development pathway can enhance translational accuracy (4–7). We assessed the performance and predictive capacity of umbilical cord derived human hematopoietic stem cell (HSC)- and human peripheral blood mononuclear cell (PBMC)- engrafted, humanised mouse models using a reference panel of recombinant mAbs with varying capacities to induce CRS in clinical settings. The reference Ab panel (NIBSC code 19/156), earlier evaluated for *in vitro* CR profile in an international collaborative study (8), comprises of six mAbs including three positive control mAbs: human anti-CD28- SA, mouse anti-CD3, human anti-CD52, manufactured according to the respective published sequences of TGN1412 (theralizumab, IgG4) (9, 10), Orthoclone OKT-3^®^ (muromonab, IgG2a) (11–13) and Campath-1H^®^ (alemtuzumab, IgG1) (14, 15), and three isotype matched negative controls: human IgG4, mouse IgG2a and human IgG1, respectively. Our results provide the first comparative evaluation of CR responses across the above engraftment models using a unified reference Ab panel. Furthermore, we discuss the utility of both *in vitro* and *in vivo* approaches for enhancing translational relevance for hazard identification and preclinical safety assessment in the clinical development process of biological medicines.

## 2 Materials and methods

### 2.1 Immune cell engraftment of NSG mice

Female NOD.Cg-Prkdc^scid^ Il2rg^tm1Wjl^/SzJ mice (NSG, stock number 005557) were purchased from the Jackson Laboratory (import arranged through Charles River Laboratories, Harlow, UK). Animals were housed in aseptic, bio-exclusion conditions in individually ventilated cages (IVCs) at a maximum of 5 animals per cage and provided autoclaved food. All animal procedures were performed in strict accordance with UK Home Office (HO) guidelines, under a licence: PP5307427 “Humanised mouse models for public health research”, granted to the host establishment by the Secretary of State for the HO which approved the experimental work. Adjustments to the housing area and environmental enrichment were made by husbandry staff based on regular welfare monitoring of the experimental animals. The experimental animals were maintained at an appropriate environmental temperature, and the rooms were subject to a 12‐hour day/night lighting cycle. Prior to inclusion in the study, animals were acclimatised to the environment and assessed for health and welfare by the named veterinary surgeon (NVS). Experimental animals were routinely monitored for changes in weight and overall condition by weight measurement and individual visual evaluations. The general health monitoring severity limit for the present study, under the animal licence is classified as moderate category. Animals showing any of the mild clinical signs in the mild category including 10% body weight loss, partial piloerection or transient hunched posture were closely monitored, under advice from the NVS and/or named animal care and welfare officer (NACWO). Animals were humanely euthanised in cases where an animal exhibited more than three mild clinical signs and no improvement was observed within 48 hours. Similarly, any experimental animal experiencing a 15% of body weight loss or two other moderate severity clinical signs was euthanised.

Mice were preconditioned with X-ray irradiation (RS 2000, Rad Source Technologies, US) using whole body irradiation at 140cGy, at least 4h before intravenous (i.v.) injection of HSCs or PBMCs. CD34^+^ cells (ID: CBC121211A, CBC121212A, CBC121101A, CBC121030A and CBC121108B) were sourced from AllCells LLC (California, USA) and identified as 1211A, 1212A, 1101A, 1030A, 1108B respectively for the experiment. Cryopreserved CD34^+^ cells were thawed and expanded for 2 days in StemSpan™ SFEM II serum-free expansion medium supplemented with StemSpan™ CD34^+^ expansion supplement in accordance with manufacturer’s instructions (STEMCELL Technologies UK Ltd., Cambridge, UK). CD34^+^ cell count for each stem cell preparation was flow cytometrically determined using BD Stem Cell reagent CD45/CD34: CD45 FITC, clone 2D1, and CD34 PE, clone 8G12 (BD Biosciences, California, USA). CD34^+^ HSCs were identified as CD45^dim^ CD34^+^. As detailed above, five CD34^+^ donor cell stocks were used for the engraftment study to account for donor variability. A total of thirty irradiated female NSG mice (five-week-old), were intravenously administered 0.15 × 10^6^ CD34^+^ cell suspension in 100 μl phosphate buffered saline, pH: 7.4 (PBS).

For the PBMC-engraftment model, cryopreserved PBMCs were obtained from the indicated commercial suppliers. Donor ID 19TL137529, 19TL326418, 19TL353633 (Lonza bioscience, California, USA) were identified as D4, D15 and D17, respectively for the experiment and 181281804C, 181181301C, 1711220135, 1801120084 and 1810110107 (STEMCELL Technologies Inc., Massachusetts, USA) identified as D1, D2, D9, D20, D38, respectively. Eight different donor PBMC preparations were utilised in the study to consider potential inter-donor variability in responses. Eight-week-old irradiated female NSG mice were intravenously injected with 20 × 10^6^ PBMC cell suspension in 100 μl PBS.

### 2.2 *In vivo* assessment of Ab efficacy and safety

Engraftment levels were determined by flow cytometry analysis as detailed in the following section 2.3. Mice were randomly grouped prior to i.v. injection with 20 μg of respective Abs (code 19/156, NIBSC-MHRA, UK) in 100 μl PBS. Cell and Ab administrations were performed under blinded conditions to minimise operator bias. For the CD34^+^ HSC engraftment model, six groups of mice were injected with either anti-CD28- SA, anti-CD3, anti-CD52 (n= 5 for each group), or their respective isotype controls IgG4, IgG2a and IgG1 (n= 4 for each group), at week 22 post CD34^+^ injection. For the PBMC engraftment model, seven groups of mice (n= 6 for each group) were injected respectively either with anti-CD28- SA, anti-CD3, anti-CD52, or their respective isotype controls IgG4, IgG2a, IgG1 or PBS at day 7 post PBMC injection. Mice were bled at 2, 4, 6 and 24h following mAb injection and plasma samples stored at -20°C until cytokine analysis. Tail bleed samples were collected using Microvette^®^ 100 Lithium heparin LH, capillary blood collection tubes (SARSTEDT AG & Co. KG, Nümbrecht, Germany) and blood cells preserved by addition of an equal volume of Streck cell preservative (Streck, Nevada, USA) for immunophenotypic analysis by flow cytometry. Plasma samples were collected by allowing natural coagulation of the blood sample, followed by centrifugation at 5000 rpm for 10 minutes. The clear supernatant was collected and stored at -20°C until further analysis.

### 2.3 Flow cytometry analysis

Human immune cell reconstitution was monitored at 9, 12, 15, 18 and 22 weeks following the injection of CD34^+^ HSC. Engrafted human cells were distinguished from host mouse cells using Abs against mouse and human CD45. PBMC engraftment was assessed at day 5 post PBMC injection. Abs used for flowcytometry were purchased from the indicated suppliers. Preserved blood sample was stained in Trucount tube (BD biosciences, UK) for cell count determination, with the following Ab panel for the HSC engraftment model: mouse CD45-perCPcy5.5 (clone 30-F11), human CD45-APC (clone HI30), CD20-FITC (clone 2H7), CD8-APC-cy7 (clone SK1), and CD56-PEcy7 (clone B159) sourced from BD biosciences, Berkshire, UK and CD3-Pacific Blue (clone UCHT1),CD4-PE (clone SK3), sourced from Biolegend (London, UK).

Flow cytometry staining for the samples from PBMC engraftment model was performed similarly in BD Trucount tubes, using the following Ab panel: mouse CD45-perCPcy5.5 (clone 30-F11) sourced from BD biosciences, Berkshire, UK and human CD45-FITC (clone 2D1), CD20-BV510 (clone 2H7), CD3-PB (clone UCHT1), CD4-PE (clone SK3), CD8-APC-cy7 (clone RPA-T8), all sourced from Biolegend (London, UK). Samples were incubated with staining Abs for 40 minutes at room temperature. Stained samples were treated with 1 ml 1x RBC Lysis buffer (diluted from 10x solution prepared in house containing 8.02 gram/ml ammonium chloride, 0.84 gram/ml sodium bicarbonate, 0.37 gram/ml disodium EDTA), added just prior to acquisition. Minimum of 10000 cell gate events were acquired on BD FACSCanto II flow cytometer (BD Biosciences, UK). Data analysis was performed with FlowJo software (version 10.8.0 or higher, Oregon, USA). Gating strategy employed for analysis is represented in **Supplementary Figure 1**.

For unsupervised clustering, uniform manifold approximation and projections (UMAPs) showing phenotypic profiles of human CD45^+^ population were generated individually for each engraftment model, from concatenated flow cytometry standard (FCS) files using UMAP_R plugin in FlowJo. The final concatenated files ready for UMAP generation were created by first compiling treatment-group labelled individual samples, followed by exporting beads and human CD45^+^ populations from the file, normalisation of total cell events per treatment group using bead counts and finally concatenation of bead-normalised treatment coded population into a single FCS file. Further details are schematically represented in **Supplementary Figures 2**, **3**. Five parameters comprising the fluorescent intensities of CD3, CD4, CD8, CD20 and CD56, were included for UMAP generation.

### 2.4 *In vitro* cytokine release assays

Cryopreserved donor PBMCs from same set of donors detailed in section 2.1 were evaluated *in vitro*. Solid phase (SP) CRA was performed by wet coating wells of Corning^®^ 96-well clear, round bottom, polypropylene, untreated microplates (Corning, #3879) purchased from Corning (Flintshire, UK). Microtiter wells were coated with 100 μl media/PBS containing mAbs (10 µg/ml; corresponding to 1 µg/well) and incubated overnight at 4°C (16–18). Unbound mAb was removed by washing wells with 1x PBS. PBMC suspension was adjusted to a concentration of 1 × 10^6^ cells/ml in RPMI containing 10% heat-inactivated foetal bovine serum (FBS) (18) and 200 μl cell suspension was added to each well. Aqueous phase (AQ) CRA using PBMCs was performed in Corning^®^ 96-well clear, round bottom tissue culture-treated microplate (Corning, #3799) purchased from Corning (Flintshire, UK). PBMCs were seeded as described above for SP-CRA and co-incubated with mAb at a final concentration of 5 µg/ml. Plates for *in vitro* CRA were incubated for 48 hours in a humidified incubator at 37°C, 5% CO_2_. Cytokine-conditioned supernatant was obtained by centrifuging the plates and samples were stored at -20°C until measurement as described below.

### 2.5 Cytokine measurement

Measurement of cytokine levels interleukin 2 (IL-2), interleukin 6 (IL-6), interleukin 10 (IL-10), interferon γ (IFN-γ), and tumour necrosis factor α (TNF-α), was performed in diluted plasma samples or culture supernatants from *in vitro* CRA, using custom made V-PLEX proinflammatory panel 1 human kit (Meso Scale Discovery, Maryland, USA) in accordance with manufacturer’s instructions. Plates were read on The MESO QuickPlex SQ 120MM, with Methodical Mind software (Meso Scale Discovery, Maryland, USA). Data analysis was performed using GraphPad Prism software (version 8.1.1, Massachusetts, USA).

Principal component analysis (PCA) of cytokine concentration was performed by first rescaling of the 5-dimensional data using sklearn.preprocessing.StandardScaler (mean= 0, standard deviation (SD)= 1), followed by the calculation of PC scores, explained variances ratios and eigenvectors using sklearn.decomposition.PCA. Numpy and Pandas packages were used for basic data handling during the process. Distance biplots showing scaled eigenvector of individual features (cytokine) and PC scores were generated using matplotlibs.pyplot and seaborn packages. A scaling factor of 4 or 5 was applied to eigenvectors for improved visualisation of vector direction. In an unscaled eigenvector_cytokine_ (x, y), the co-ordinates represent the correlation coefficients between standardised concentration of the named cytokine and PC1 score in the case of x or PC2 score in the case of y.

### 2.6 Secretome analysis

Select plasma samples from treated mice in each engraftment model, chosen by randomisation were additionally evaluated using Olink Reveal kit (Olink Proteomics AB, Uppsala, Sweden) in accordance with manufacturer’s instructions. In brief, the Olink proximity extension assay (PEA) uses oligonucleotide conjugated Abs for target protein recognition. A subsequent polymerase chain reaction (PCR) step amplifies bound conjugates for specific protein quantification. Unique conjugate sequences within the resulting PCR library allow the match of amplification intensity to target proteins during downstream analysis. Pooled Olink Reveal library quality was assessed using Biolanalyser 2100 instrument with DNA High Sensitivity kit (Agilent, USA). Sequencing was performed on a NextSeq 2000 platform using a P4 50-cycle kit (Illumina, California, USA) at 24 single-end reads, with a custom Olink sequencing recipe, as per manufacturer’s instructions.

Raw sequencing reads were converted into protein counts using Olink ngs2counts tool (v5.1.0). Olink NPX Map software (v1.1.3) was then used to review data quality and normalise the data, converting the counts to normalised protein expression (NPX) units. NPX is a relative measure of protein expression in log_2_ scale reflecting the amplification intensities during the PCR step. Linear mixed-effects regression (LMER) analysis and *post-hoc* analysis comparing secretomes between positive control test Ab and matched isotype control groups were performed using the R-package (OlinkAnalyze, v4.3.1). A threshold of adjusted p-value < 0.1 was applied to define statistical significance in protein expression, considering the limited sample size (n=1–2) per condition and the exploratory nature of the analysis.

LMER analysis outputs were then exported for downstream analysis and graph generations. Python packages Pandas and Numpy were used for further handling and downstream analysis of LMER outputs. Log_2_ fold change of differential expressions were calculated by NPX_positive test Ab_ - NPX_isotype control_. Python packages matplotlib.pyplot, matplotlib_venn and seaborn were used in combination for Venn diagrams, swarm plots and heatmap generation. Subcellular locations of target protein were determined by mapping protein accession code to the universal protein (UniProt) database, using request package. Secreted and known ectodomain cleaved proteins were classified as “secreted”, while proteins from endoplasmic reticulum/golgi, mitochondria, or with unknown subcellular locations were combined into the “other compartment” category.

### 2.7 Statistical analysis

All data are derived from biological replicates involving independent donors as indicated for each experimental model. Except for secretome data (Section 2.6), statistical analyses were performed on log_10_ transformed data using Graph Pad prism software (version 10.4.1, MA, USA). Time course analysis of CR was performed using two-way ANOVA using Tukey’s test with 95% confidence to counteract for multiple comparisons. Cell analysis after treatment was performed using one-way ANOVA followed by Šídák’s *post-hoc* test with 95% confidence interval to counteract for multiple comparisons. Mean results were calculated for each response and treatment method. Statistical significance is represented as p >0.05 (not significant; ns), p ≤ 0.05 (*), p ≤ 0.01 (**), p ≤ 0.001 (***), and p ≤ 0.0001 (****).

## 3 Results

### 3.1 Efficient immune reconstitution in HSC and PBMC- engrafted NSG mice

We evaluated two models of immune reconstitution using the unified reference Ab panel, 19/156. Human HSC- and PBMC-engrafted humanised mouse models were generated as described above.

A preliminary study was conducted to determine the optimal CD34^+^ cell number for consistent human cell engraftment and effective immune cell reconstitution, using indicated numbers of CD34^+^ HSCs (data not shown/**Supplementary Figure 4**). Human CD20^+^ B-cell numbers appear early, peaking at weeks 9 and 18 for the higher CD34^+^ cell dose groups, while CD3^+^ T-cells begin to emerge at week 12 for both doses. Mice given the lowest dose exhibited delayed humanisation with very low CD3^+^ T-cells by week 21. The medium dose (0.15 × 10^6^ CD34^+^ cells) that consistently produced a good level of engraftment (~800 human CD45^+^ cells/μl blood) by week 21 was used for further experiments (**Supplementary Figure 4**).

**Figure 1:**
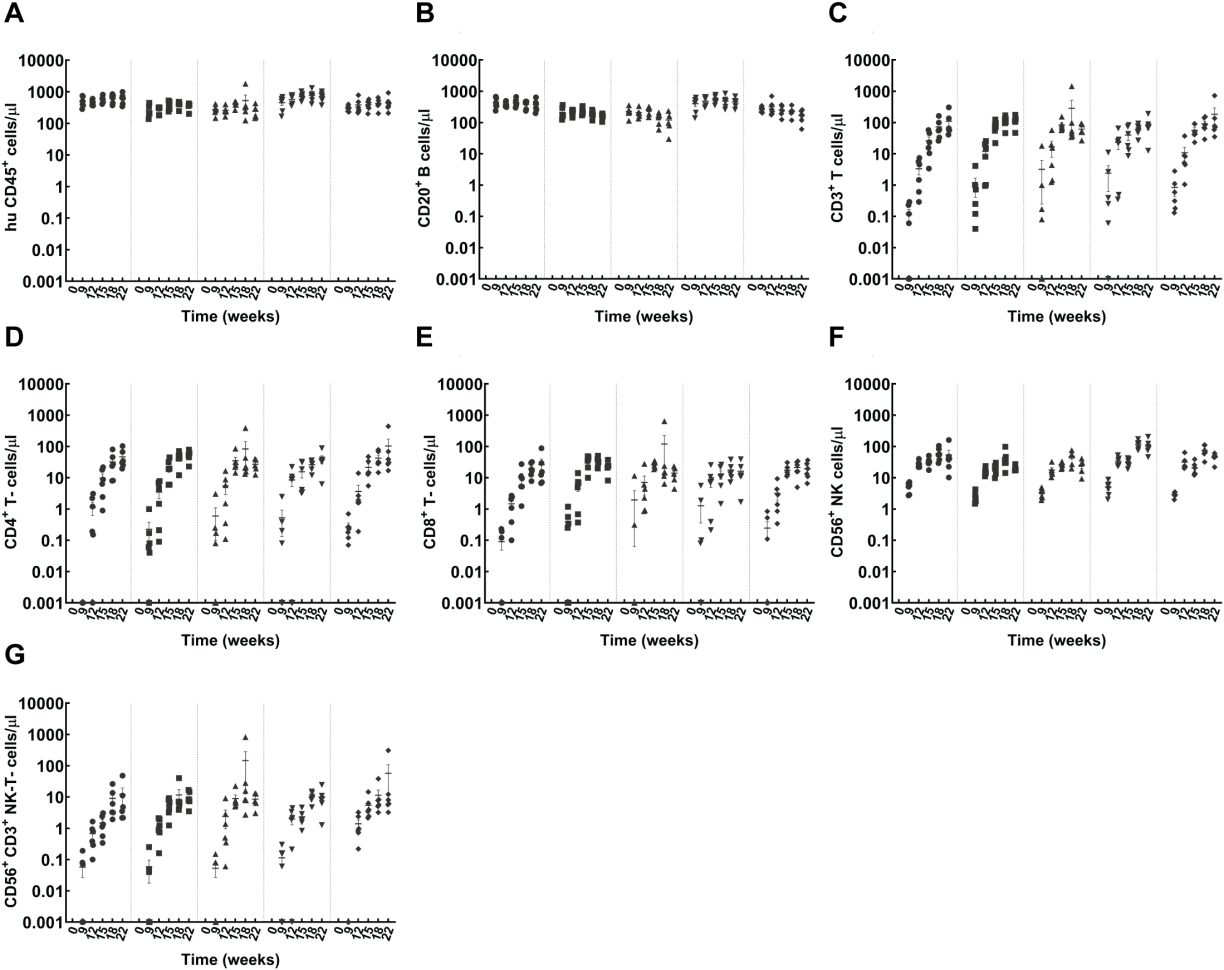
Cell engraftment kinetics in CD34^+^ HSC-engrafted NSG mice. CD34^+^ cells from individual donors were used for mouse engraftment. Mice were intravenously administered 100 μl of 0.15x10^6^ CD34^+^ cells in PBS from the indicated donors: CB121211A (•), CB121212A (▪), CB121101A (▴), CB121030A (▾), CB121108B (♦). **(A)** Total human lymphocyte (hu CD45^+^); **(B)** B cell (CD20^+^); **(C)** T- cell (CD3^+^); **(D, E)** CD4^+^ (CD3^+^, CD4^+^), and CD8^+^ (CD3^+^, CD4^+^) T- cell, respectively; **(F)** NK cell (CD56^+^); and **(G)** NK-T- cell (CD56^+^, CD3^+^) counts for each donor (left to right, separated by dotted lines) determined using BD Trucount tubes, from tail bleed samples collected at indicated time points of 9, 12, 15, 18 and 22 weeks post HSC injection are represented.

**Figure 2:**
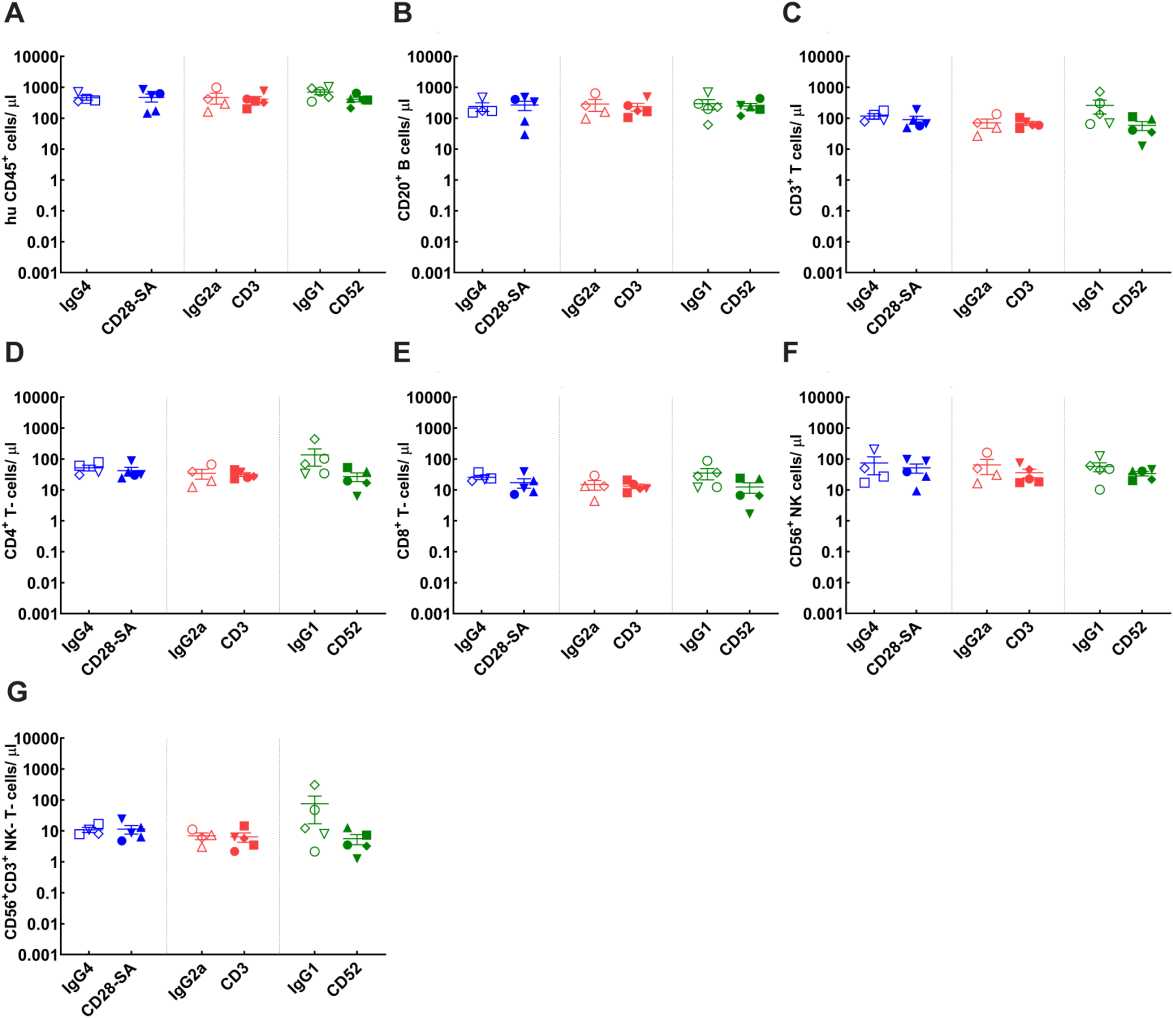
Pretreatment pattern of immune cell reconstitution and donor distribution for each experimental group of CD34^+^ HSC- engrafted NSG mice. Mice engrafted with CD34^+^ HSCs from indicated donors CB121211A (•), CB121212A (▪), CB121101A (▴), CB121030A (▾), CB121108B (♦) were randomly assigned for treatment with either the positive control test Ab (filled symbols): anti-CD28- SA (blue), anti-CD3 (red) or anti-CD52 (green); or respective isotype control Abs (hollow symbols): IgG4 (blue), IgG2a (red) or IgG1 (green). **(A)** Total human lymphocyte (hu CD45^+^); **(B)** B cell (CD20^+^); **(C)** T- cell (CD3^+^); **(D, E)** CD4^+^ (CD3, CD4^+^), and CD8^+^ (CD3, CD4^+^) T- cell, respectively; **(F)** NK cell (CD56^+^); and **(G)** NK-T- cell (CD56^+^, CD3^+^) counts for each experimental group, determined using BD Trucount tubes, from tail bleed samples collected prior to Ab administration (week 22) are depicted.

CD34^+^ cells from five indicated donors were used for immune reconstitution for the main experimental study. Tail bleed samples collected at indicated time points were used to assess indicated immune subsets post engraftment. Representative gating strategy used for flow cytometry quantification is presented in **Supplementary Figures 1A-F**. The level of human cell engraftment was monitored from week 9 to week 22 following CD34^+^ HSC injection. The overall pattern and kinetics of engraftment were consistent across all tested donors; however, there was variability among donors in absolute cell counts, post reconstitution (**Figure 1**). CD20^+^ B-cell engraftment initiated early by week 9 with an average cell count 306 cells/μl, peaking at week 15 with a mean value of 344 cells/μl, then lowering to a mean value of 261 cells/μl by week 22 (**Figure 1**, **Supplementary Figure 5**). On the other hand, CD3^+^ T-cell count steadily increased starting from an average value of 2 cells/μl at week 9 to an average value of 112 cells/μl by week 22. Consistent with the expected reference value range for healthy human donors (19), the observed mean CD4^+^/CD8^+^ T-cell ratio in HSC-engraftment model was 2.8 by week 22 (**Figure 1**, **Supplementary Figure 5**). However, donor 1101A, exhibited a slight decrease in overall T-cell and CD8^+^ T-cell numbers at week 22. The average proportions of various immune cell populations among human CD45^+^ cells at week 22 were as follows: B-cells (54%), T-cells (23%), CD4^+^ T-cells (11%), CD8^+^ T-cells (4%), NK cells (11%), and NK-T cells (4%) (**Figure 1**, **Supplementary Figure 5**, **Supplementary Table 1A**). These results show that the HSC model predominantly facilitated B-cell differentiation, along with various other immune cell subsets including T-cells, NK and NK-T cells. Prior to the administration of test Abs at week 22, mice were randomised to account for HSC- donor variability and the average pre-treatment immune cell distribution in each experimental group is represented in **Figure 2** and **Supplementary Table 1A**.

**Figure 3:**
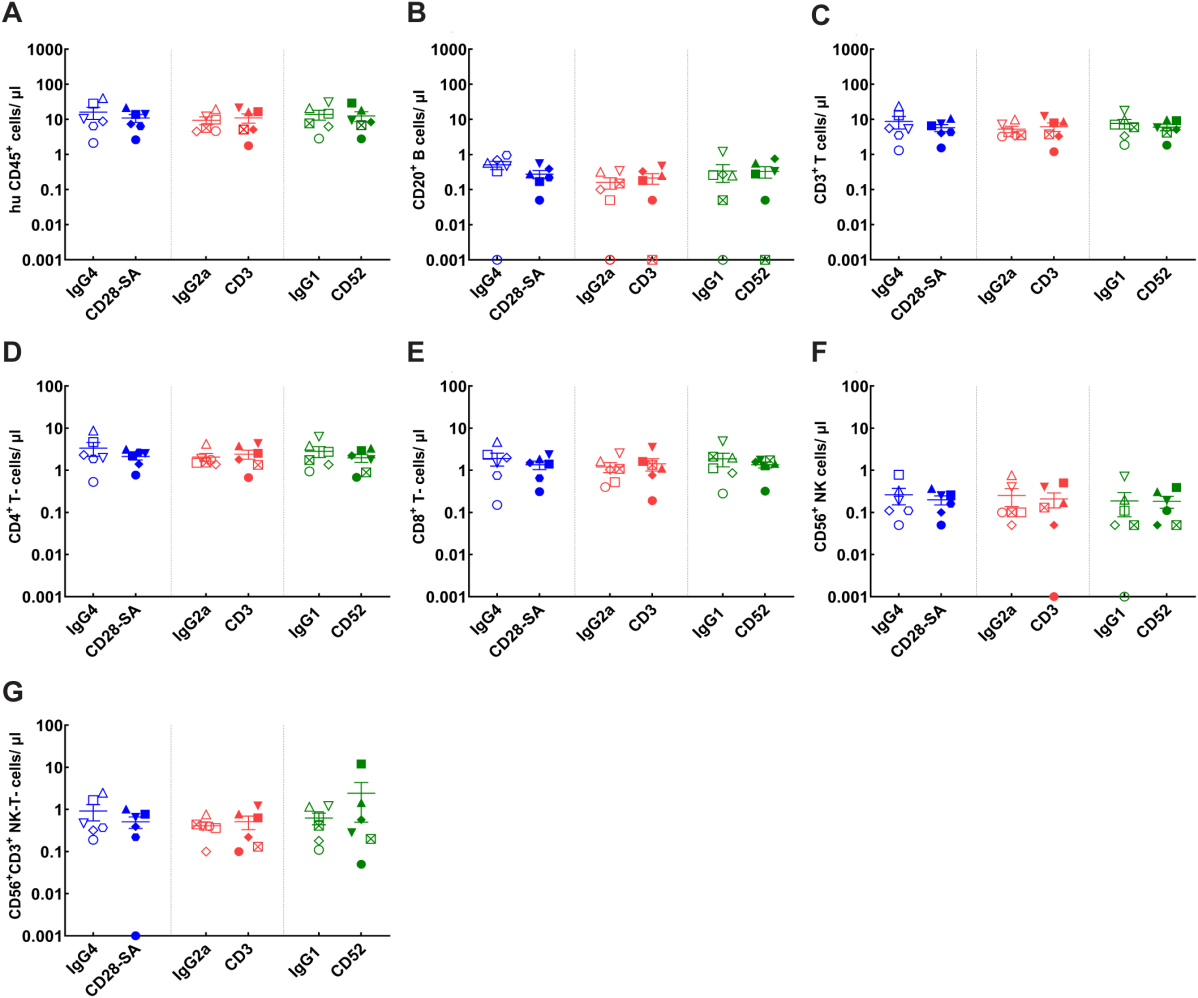
Pretreatment immune cell reconstitution profiles and donor distribution across experimental groups of PBMC-engrafted NSG mice. Mice engrafted with PBMCs from individual donors were randomly selected for treatment with either the positive control test (filled symbols): anti-CD28- SA (blue), anti-CD3 (red) or anti-CD52 (green); or matched isotype control Abs (hollow symbols): IgG4 (blue), IgG2a (red) or IgG1 (green). Each individual donor D1 (♦), D2 (▾), D4 (•), D9 (▪), D10 (▴), D17 (⬣), D20 (☒) is marked by the indicated symbol. Total human lymphocyte (hu CD45^+^); **(B)** B cell (CD20^+^); **(C)** T- cell (CD3^+^); **(D, E)** CD4^+^ (CD3^+^, CD4^+^), and CD8^+^ (CD3^+^, CD4^+^) T- cell, respectively; **(F)** NK cell (CD56^+^); and **(G)** NK-T- cell (CD56^+^, CD3^+^) counts for each experimental group, determined using BD Trucount tubes, from tail bleed samples collected prior to Ab administration (day 5) are depicted.

A total of eight distinct donor PBMC preparations were used for immune reconstitution for the main study. NSG mice were engrafted across two independent experiments for evaluation of the PBMC-engraftment model. Human immune cell subset reconstitution was assessed at day 5 post PBMC injection. Representative gating strategy used for flow cytometry quantification following PBMC engraftment is presented in **Supplementary Figures 1G– L**. In contrast to the HSC model, the PBMC model showed minimal CD20^+^ B-cell engraftment with an average cell count 0.3 cells/μl, while CD3^+^ T-cells formed the major proportion of human CD45^+^ cells with an average value of 7 cells/μl by day 5 (**Figure 3**, **Supplementary Table 2A**). The mean CD4^+^/CD8^+^ T-cell ratio was 1.6, which is lower than the ratio observed in the HSC model, but within the expected reference range for healthy donors. The PBMC model also supported low levels of NK and NK-T-cell engraftment. Interestingly, the ratio of NK/NK-T cells was lower (0.2) in the PBMC model when compared to the HSC model (2.5), where a larger relative expansion of NK cells was observed by week 22. These findings indicate that the PBMC model achieves an engraftment pattern predominated by CD3^+^ T-cells and while the HSC model predominantly supports B-cell reconstitution, all assessed immune cell subsets are efficiently represented.

### 3.2 Treatment with reference Ab panel 19/156, induced characteristic cytokine patterns in HSC- versus PBMC- engrafted humanised NSG mice

Following human cell engraftment (week 22 for HSC- and day 5 for PBMC-engraftment model), mice were grouped randomly for treatment with the reference Ab panel, 19/156. Following treatment, mice were bled at indicated time points and cytokine profiles assessed by MSD assay.

**Figure 4:**
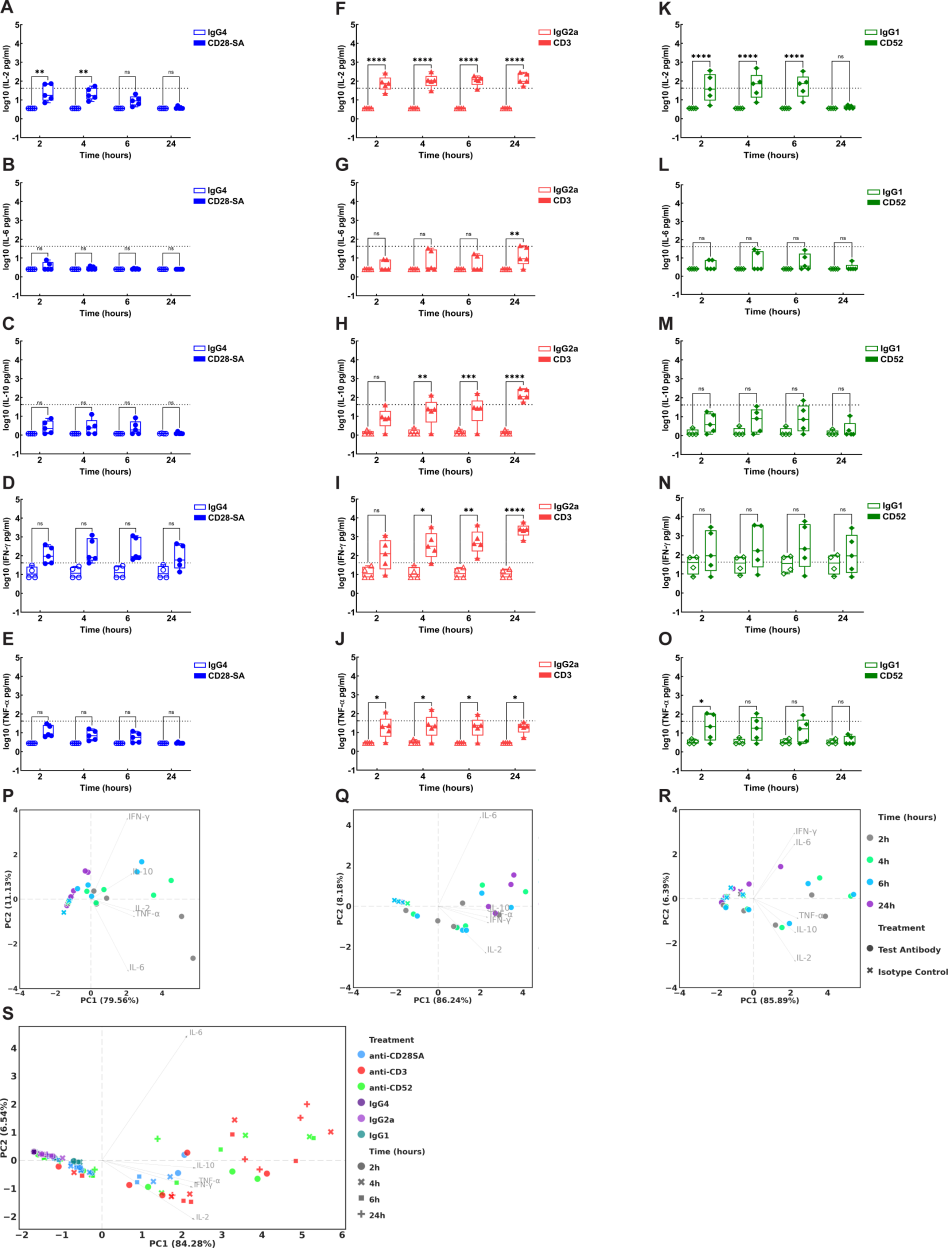
Cytokine concentrations in CD34^+^ HSC-engrafted NSG mice following administration of either the positive control test or the corresponding isotype control Ab. Mice engrafted with CD34^+^ HSC were treated intravenously with either 20 μg positive control test (filled symbols): **(A–E)** anti-CD28- SA (blue), **(F–J)** anti-CD3 (red) or **(K–O)** anti-CD52 (green); or respective isotype control Abs (hollow symbols): **(A–E)** IgG4 (blue); **(F–J)** IgG2a (red); or **(K–O)** IgG1 (green). Quantification of cytokine level for **(A, F, K)** IL-2; **(B, G, L)** IL-6; **(C, H, M)** IL-10; **(D, I, N)** IFN-γ and **(E, J, O)** TNF-α in plasma samples from HSC- engrafted mice collected at 2h, 4h, 6h and 24h post Ab administration was performed by MSD multiplex assays following manufacturer’s instructions. The plots represent log_10_ transformed values for level of each indicated cytokine in picograms/ml. Statistical analysis was performed using GraphPad Prism version 10.4.1, as detailed in Materials and Methods. **(P-S)** Principal component analysis (PCA) was performed as detailed in Materials and Methods. PCA plots derived from cytokine concentration display experimental data from HSC-engrafted mice following treatment with **(P)** anti-CD28- SA, **(Q)** anti-CD3, **(R)** anti-CD52, or **(S)** combined data across all groups at indicated time points. The coordinates of arrowheads in the PCA biplots are 5-fold scaled eigenvectors. The unscaled eigenvectors represent the correlation of PC scores to the corresponding features. p ≤ 0.05 (*), p ≤ 0.01 (**), p ≤ 0.0001 (****), not significant (ns).

Cytokine profiles observed in response to Ab treatment in CD34^+^ HSC-engrafted NSG mice are represented in **Figure 4** and **Supplementary Table 3**. A pronounced early induction of IL-2 was observed in mice treated with anti-CD28- SA, initiated by 2h (~10-fold increase compared to IgG4) and declining gradually by 6 hours (**Figure 4A**). In contrast, induction levels of IL-6, IL-10, and TNF-α remained minimal following anti-CD28- SA administration (**Figures 4B, C, E**). IFN-γ levels increased gradually, peaking at 4-6h, post anti-CD28- SA treatment (**Figure 4D**). Notably, the levels observed for all tested cytokines, except for IL-2, did not reach statistical significance (**Figures 4B–D**). Anti-CD3 treatment induced significant release of all the assessed cytokines including IL-2, IL-6, IL-10, IFN-γ and TNF-α compared to the isotype control IgG2a (**Figures 4F–J**). Both IL-2 and TNF-α were observed to be induced early from 2h post anti-CD3 treatment (29-fold and 13-fold increase respectively) and were either sustained or showed only a slight decrease by 24h (**Figures 4F, J**). Interestingly, while IL-6 induction was observed from 2h post treatment, the levels did not reach statistical significance until 24h post anti-CD3 treatment (~8-fold increase) (**Figure 4G**). IL-10 and IFN-γ were significantly induced beginning at 4h post anti-CD3 treatment and the levels steadily increased over time, reaching maximum values corresponding to ~122-fold and ~224-fold increase respectively by 24h (**Figures 4H, I**). Anti-CD52 treatment induced significant IL-2 and TNF-α release (~31-fold and 14-fold increase respectively) within 2h compared to the IgG1 isotype control (**Figures 4K, O**). Comparable pattern of early IL-2 induction was observed in mice treated with either anti-CD3 or anti-CD52. However, while IL-2 levels were sustained for up to 24h following anti-CD3 treatment (**Figure 4F**), anti-CD52 treatment resulted in rapid IL-2 induction by 2h (32-fold), that was sustained till 6h, declining sharply by 24h (**Figure 4K**). IL-6, IL-10, and IFN-γ exhibited comparable patterns of increase, beginning as early as 2h following anti-CD52 administration and subsequently declining by 24h (**Figures 4L–N**). However, the measured concentrations of these cytokines did not achieve statistical significance. TNF-α was significantly and transiently induced early by 2h following anti-CD52 treatment (~14-fold) (**Figure 4O**).

**Figure 5:**
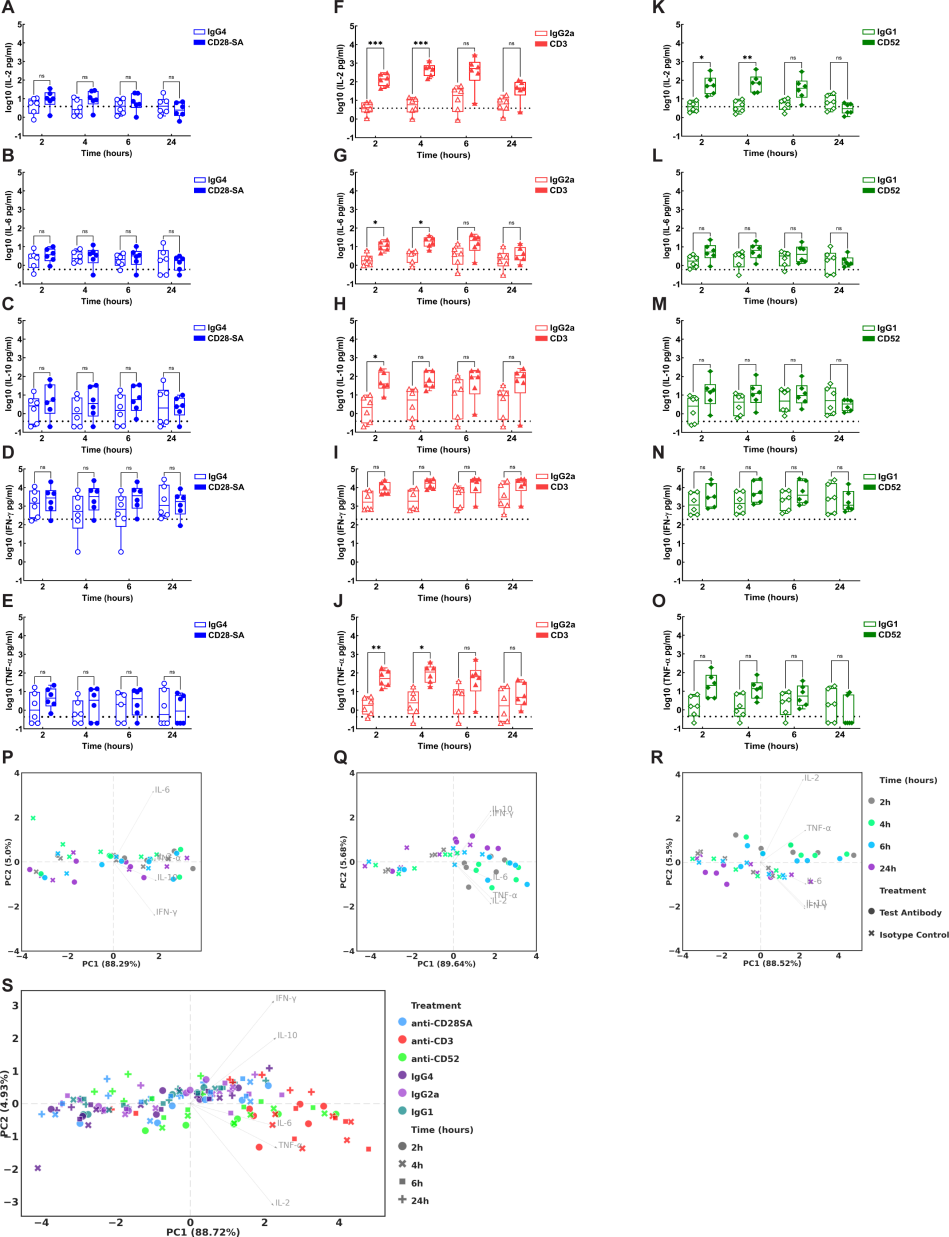
Cytokine levels in PBMC-engrafted NSG mice following treatment with positive or isotype control Abs. PBMC-engrafted mice engrafted received intravenous administration of either 20 μg positive control test (filled symbols): **(A–E)** anti-CD28- SA (blue), **(F–J)** anti-CD3 (red), or **(K–O)** anti-CD52 (green); or corresponding isotype control Abs (hollow symbols): **(A–E)** IgG4 (blue); **(F–J)** IgG2a (red); or **(K–O)** IgG1 (green). Quantification of cytokine level for **(A, F, K)** IL-2; **(B, G, L)** IL-6; **(C, H, M)** IL-10; **(D, I, N)** IFN-γ and **(E, J, O)** TNF-α in plasma samples from engrafted mice collected at 2h, 4h, 6h and 24h post Ab administration was performed by MSD multiplex assays following manufacturer’s instructions. The plots show log_10_-transformed cytokine levels in picograms/ml. Statistical analyses were conducted with GraphPad Prism version 10.4.1, as described in the Materials and Methods section. **(P-S)** PCA plots derived from cytokine concentrations represent experimental data from PBMC- engrafted mice collected at indicated time points, following treatment with **(P)** anti-CD28- SA, **(Q)** anti-CD3, **(R)** anti-CD52 or **(S)** combined data for all treatment groups. The coordinates of arrowheads in the PCA biplots are 5-fold scaled eigenvectors. The unscaled eigenvectors represent the correlation of PC scores to the corresponding features. p ≤ 0.05 (*), p ≤ 0.01 (**), p ≤ 0.001 (***), not significant (ns).

Cytokine induction following Ab treatment in PBMC engrafted-NSG mice are presented in **Figure 5** and **Supplementary Table 4**. Treatment with anti-CD28- SA only showed very modest IL-2, IL-6, IL-10 and TNF-α induction that did not reach statistical significance (**Figures 5A–E**). Anti-CD3 treatment on the other hand, induced significant release of these cytokines. IL-2 release was initiated early by 2h, reaching an approximately 35-fold increase by the 6h time point, before reducing by 24h (**Figure 5F**). IL-6 induction was comparatively low but significantly detected at 4h and 6h respectively (~4-6-fold increase) (**Figure 5G**). IL-10 levels showed an early significant increase at 2h post anti-CD3 treatment and remained steady with an approximately 10-fold increase by 24h (**Figure 5H**). TNF-α levels similarly showed a significant increase at 2h and 4h, peaking with a 26-fold increase, and declining by 24h post treatment (**Figure 5J**). Similar to the profile observed with anti-CD3, treatment with anti-CD52 showed significant and early IL-2 induction (21-fold), peaking by 4h (**Figure 5K**). Although incremented levels of IL-6, IL-10 and TNF-α were observed following anti-CD52 treatment, the levels did not reach statistical significance (**Figures 5L–O**). Notably, high IFN-γ levels (≥2000 pg/ml) were observed for all treatment groups, including control mice injected with respective isotype controls or PBS alone, in the PBMC-engraftment model. Though the IFN-γ levels, during the first 6h, were approximately 3-5-fold higher with the test Abs compared to their respective isotype controls, these increases did not reach statistical significance (**Figures 5D, I, N**). Overall, these results indicate that treatment with each Ab in the reference panel produces a distinct cytokine profile displaying varying kinetics and patterns in HSC- versus PBMC- engrafted humanised mice (**Figures 4**, **5**).

To gain an objective overview of the cytokine profile in response to each Ab, dimensionality reduction by PCA was conducted. Across both engraftment models, all assessed cytokines exhibited strong association, as indicated by the high percentage of variances explained by PC1 (ranging from 79.56% to 89.64%). and the nearly identical X-components of eigenvectors (0.4 to 0.48) across all the eight PCAs (**Figures 4P–S**, **5P–S**). On the contrary, the Y-components of eigenvectors were variable (ranging from -0.7 to 0.7) across PCAs, with PC2 explaining between 4.93% to 11.13% of the variance, indicating that some samples had profiles dominated by specific cytokines though these occurred with modest magnitude or frequency. In comparison to PBMC-engrafted mice (**Figures 5P–S**), the HSC-engrafted NSG mice exhibit low sample variation among isotype controls and better distinction between the positive test Ab and the isotype controls, while the background variance observed in the PBMC-engraftment model was higher (**Figures 4P–S**). Apart from the isotype controls in HSC-engrafted NSG mice, all samples exhibit considerable variations as illustrated by the loose clustering, potentially attributable to donor variability. Post-treatment time was a key factor influencing cytokine profiles, as observed in anti-CD28- SA treated HSC-engrafted NSG mice (**Figure 4P**), and in both engraftment models, post anti-CD3 treatment (**Figures 4Q**, **5Q**). Additionally, all treatment-control pair datasets were incorporated within the same PCA space for a parallel comparison (**Figures 4S**, **5S**). Comparable to the results highlighted earlier, the treatment-control distinction in anti-CD28SA treatment was weak in HSC engrafted NSG and negligible in PBMC engrafted NSG mice, while anti-CD3 treatment consistently triggered stronger CR than isotype control in both models. Anti-CD52 similarly lead to CR in both engraftment models, though the treatment-control differences were comparatively weaker in the PBMC engraftment model.

### 3.3 Immune cell composition post treatment with reference Ab panel 19/156 in HSC- versus PBMC- engrafted humanised NSG mice

**Figure 6:**
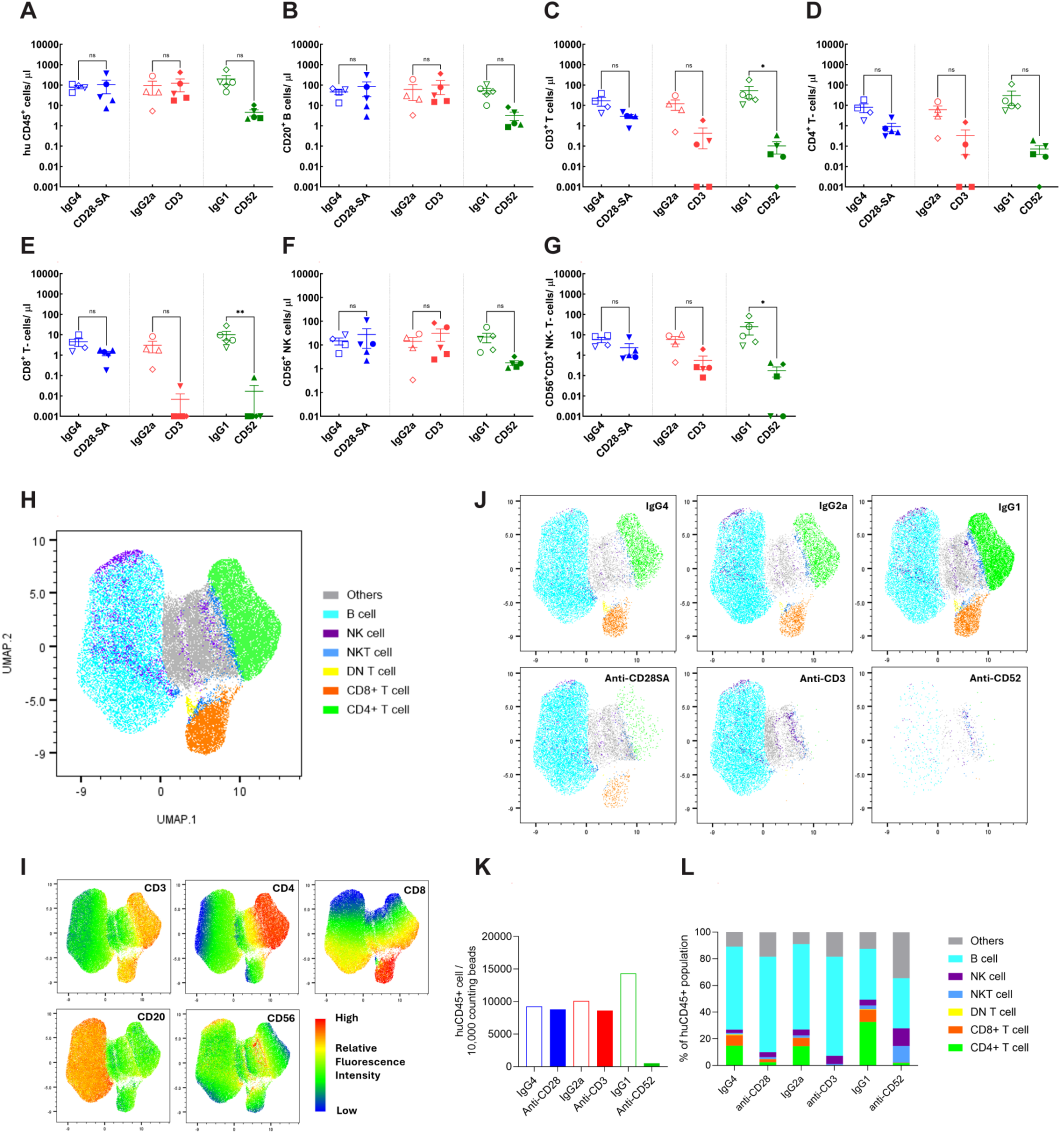
Immune cell profiles in CD34^+^ HSC- engrafted NSG mice after administration of positive or isotype control Abs. Mice engrafted with CD34^+^ HSCs from indicated donors CB121211A (•), CB121212A (▪), CB121101A (▴), CB121030A (▾), CB121108B (♦) were administered either the positive control test (filled symbols): anti-CD28- SA (blue), anti-CD3 (red) or anti-CD52 (green); or matched isotype control Ab (hollow symbols): IgG4 (blue), IgG2a (red) or IgG1 (green). **(A)** Total human lymphocyte (hu CD45^+^); **(B)** B cell (CD20^+^); **(C)** T- cell (CD3^+^); **(D, E)** CD4^+^ (CD3^+^, CD4^+^), and CD8^+^ (CD3^+^, CD4^+^) T- cell, respectively; **(F)** NK cell (CD56^+^); and **(G)** NK-T- cell (CD56^+^, CD3^+^) counts for each experimental group, determined using BD Trucount tubes, from terminal cardiac bleed samples collected post treatment are depicted. **(H)** Uniform Manifold Approximation and Projection (UMAP) derived from the expression levels of the indicated five phenotypic markers, CD3, CD4, CD8, CD20 and CD56. **(I)** Plot illustrating the indicated cell types. **(J)** Plots representing pooled data for the indicated treatment groups. **(K)** Bar graph representing relative human CD45^+^ cell counts normalised to 10,000 counting beads, determined for each treatment group. **(L)** Relative cellular composition for each treatment group derived based on UMAP clusters. p ≤ 0.05 (*), p ≤ 0.01 (**), not significant (ns).

**Figure 7:**
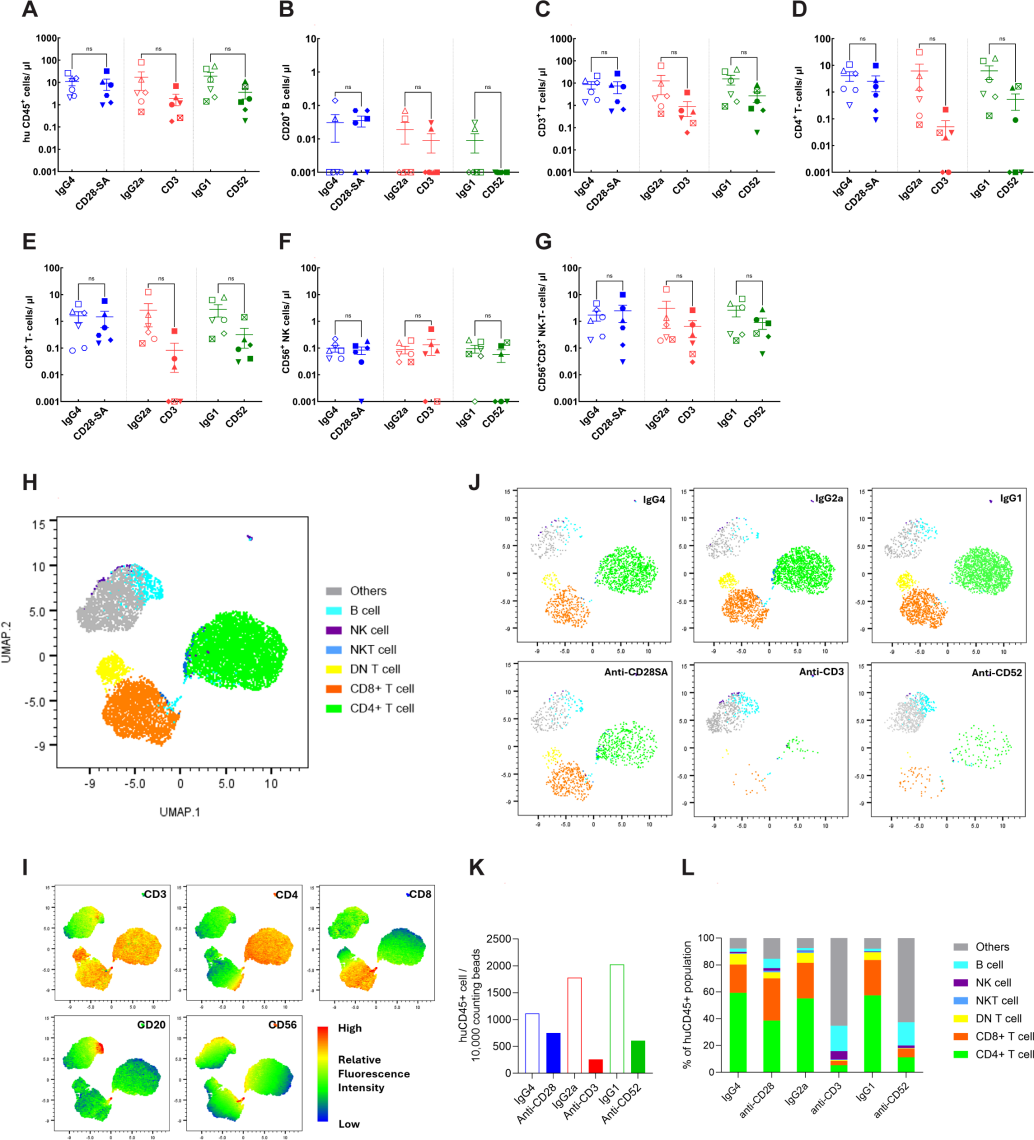
Immune cell profiles in PBMC engrafted NSG mice after administration of positive or isotype control Abs. Mice engrafted with PBMC from indicated donors 1 (♦), 2 (▾), 4 (•), 9 (▪), 10 (▴),17 (⬣), 20 (☒) were administered either the positive control test (filled symbols): anti-CD28- SA (blue), anti-CD3 (red) or anti-CD52 (green); or matched isotype control Ab (hollow symbols): IgG4 (blue), IgG2a (red) or IgG1 (green). **(A)** Total human lymphocyte (hu CD45^+^); **(B)** B cell (CD20^+^); **(C)** T- cell (CD3^+^); **(D, E)** CD4^+^ (CD3, CD4^+^), and CD8^+^ (CD3^+^, CD4^+^) T- cell, respectively; **(F)** NK cell (CD56^+^); and **(G)** NK-T- cell (CD56^+^, CD3^+^) counts for each experimental group, determined using BD Trucount tubes, from terminal cardiac bleed samples collected post treatment are depicted. **(H)** UMAP derived from the expression levels of the indicated five phenotypic markers, CD3, CD4, CD8, CD20 and CD56. **(I)** Plot illustrating the indicated cell types. **(J)** Plots representing pooled data for the indicated treatment groups. **(K)** Bar graph representing relative human CD45^+^ cell counts normalised to 10,000 counting beads, determined for each treatment group. **(L)** Relative cellular composition for each treatment group derived based on UMAP clusters. not significant (ns).

Human immune cell proportions were evaluated at the conclusion of the study, at 24h post Ab treatment for the HSC- (**Figure 6**, **Supplementary Table 1B**) and PBMC- engraftment models (**Figure 7**, **Supplementary Table 2B**). Cell enumeration following Ab treatment was performed in terminal cardiac bleed samples from HSC- engrafted mice. Treatment with anti-CD28- SA did not cause significant differences in human immune cell populations and although a modest decrease in mean values for overall human CD3^+^ T-cell and CD4^+^ and CD8^+^ T-cell counts was observed following anti-CD28- SA treatment, the values did not reach statistical significance (**Figure 6**, **Supplementary Table 1B**). Anti-CD3 treatment induced a similar but greater and significant pattern of reduction for CD3^+^ T-cell, CD4^+^, CD8^+^ T-cell counts (**Figures 6C–E**), while the modest reduction in NK T-cell counts did not reach statistical significance (**Figure 6G**). There were no reductions in CD20^+^ B-cell or CD56^+^ NK cell counts following either anti-CD28- SA or anti-CD3 treatment (**Figures 6B, F**). On the other hand, anti-CD52 treatment in HSC-engrafted mice, caused significant reduction all the indicated human cell subsets (**Figures 6A–G**).

Cell enumeration was performed in terminal cardiac and tail bleed samples from PBMC- engrafted mice. For cell count comparisons, evaluations from cardiac bleed samples are represented. Anti-CD28- SA treatment did not result in significant differences in any of the assessed human immune cell populations (**Figure 7**, **Supplementary Table 2B**). Administration of anti-CD3 induced a discernible, yet statistically insignificant reduction for overall CD3^+^ T-cell counts, however CD4^+^ and CD8^+^ T-cell counts were significantly reduced (**Figures 7C–E**). CD20^+^ B-cell and CD56^+^ NK cell counts showed no statistically significant changes in the already low numbers observed in the PBMC-engraftment model (**Figures 7B, F**). Anti-CD52 treatment in PBMC- engrafted mice, caused apparent reduction in overall human cell subsets, though, only the differences in CD4^+^ T-cell counts achieved statistical significance. CD20^+^ B-cell and CD56^+^ NK cell counts remained similar, both of which were already low in the PBMC- engraftment model (**Figures 7B, F**).

In order to objectively assess immune cell patterns post-treatment and categorise cell subsets based on intrinsic properties without manual gating bias, unsupervised clustering was additionally performed for both engraftment models (**Figures 6H–L**, **7H–L**). By dimensionality reduction using UMAP, the five selected dimensions: CD3, CD4, CD8, CD20 and CD56, of the compiled data (**Supplementary Figure 2**) from each engraftment model was embedded into a two-dimensional space with coordinates (UMAP1, UMAP2). Individual markers with low frequency or stain index, such as CD56 in HSC-engrafted mice, and CD56 and CD20 in PBMC- engrafted mice (**Figures 6I**, **7I**, **Supplementary Figure 3**), however failed to cluster distinctly. This could be an inherent limitation of one-step dimensionality reduction for data with rare cell populations. Traditional gating strategy was therefore applied for these markers as illustrated in **Supplementary Figure 3**.

Considering the embeddings as representations of immune cell populations, clear inter-model differences could be visualised (**Figures 6H, L****;** **7H, L**). The HSC-engraftment model showed a CD20^+^ B-cell- dominant profile with B-cells accounting for ~50-70% of total human CD45^+^ population (**Figures 6H, L**). PBMC- engraftment model, on the other hand, displayed a CD3^+^ T-cell-dominant pattern with T-cells comprising ~70-80% of the total human CD45^+^ population. CD56^+^ NK and CD56^+^CD3^+^ NK-T-cells were relatively rare in both engraftment models, accounting for ~5% in the HSC-engrafted control mice, while being barely detectable in PBMC-engrafted control mice. Overall, normalised total human CD45^+^ counts in isotype control mice from the HSC-engraftment model were ~5–10 fold higher compared to controls in the PBMC-engraftment model (**Figures 6K**, **7K**). The PBMC-model also demonstrated a higher engraftment variability among the isotype control groups (**Figure 7K**).

By grouping events based on treatment, clear cellular differences between the positive and isotype control Ab pairs could be observed. Anti-CD28- SA, targeting the T-cell co-stimulatory receptor, was found to lead to an approximately 75% decrease of T-cells in HSC-engraftment model (**Figures 6H–L**), while the depletion effect was minimal for the PBMC-engraftment model (**Figures 7H–L**). Anti-CD3 treatment achieved an effective clearance of T-cells in both engraftment models. Consistent with the known cytolytic feature of the Ab, anti-CD52 treatment resulted in a pronounced depletion of the majority of human CD45^+^ cells in the HSC-engraftment model and comparatively weak and T-cell biased depletion in the PBMC-engraftment model. Overall, while both unsupervised clustering and conventional gating showed similar trends, unsupervised clustering provided improved visualisation of treatment-control differences that may have biological implications but failed to reach statistical significance in conventional analyses (**Figures 6**, **7**).

### 3.4 Secretome analysis of mice engrafted with HSCs or PBMCs reveals both shared and distinct molecular signatures

Cellular profile and cytokine analyses (discussed above), showed that each Ab in the reference panel, 19/156, elicited distinct patterns of cell depletion and CR. To gain additional insight, we conducted an exploratory analysis on the secretome using the Olink Reveal panel, developed as a proteomics platform for the identification of ~1034 human proteins. This proof-of-concept study was performed with one to two randomly selected plasma samples from each engraftment model and treatment condition. By compiling differentially expressed proteins (DEPs) across indicated timepoints, the analysis allowed the identification of overall molecular signatures associated with a specific Ab treatment in each engraftment model.

**Figure 8:**
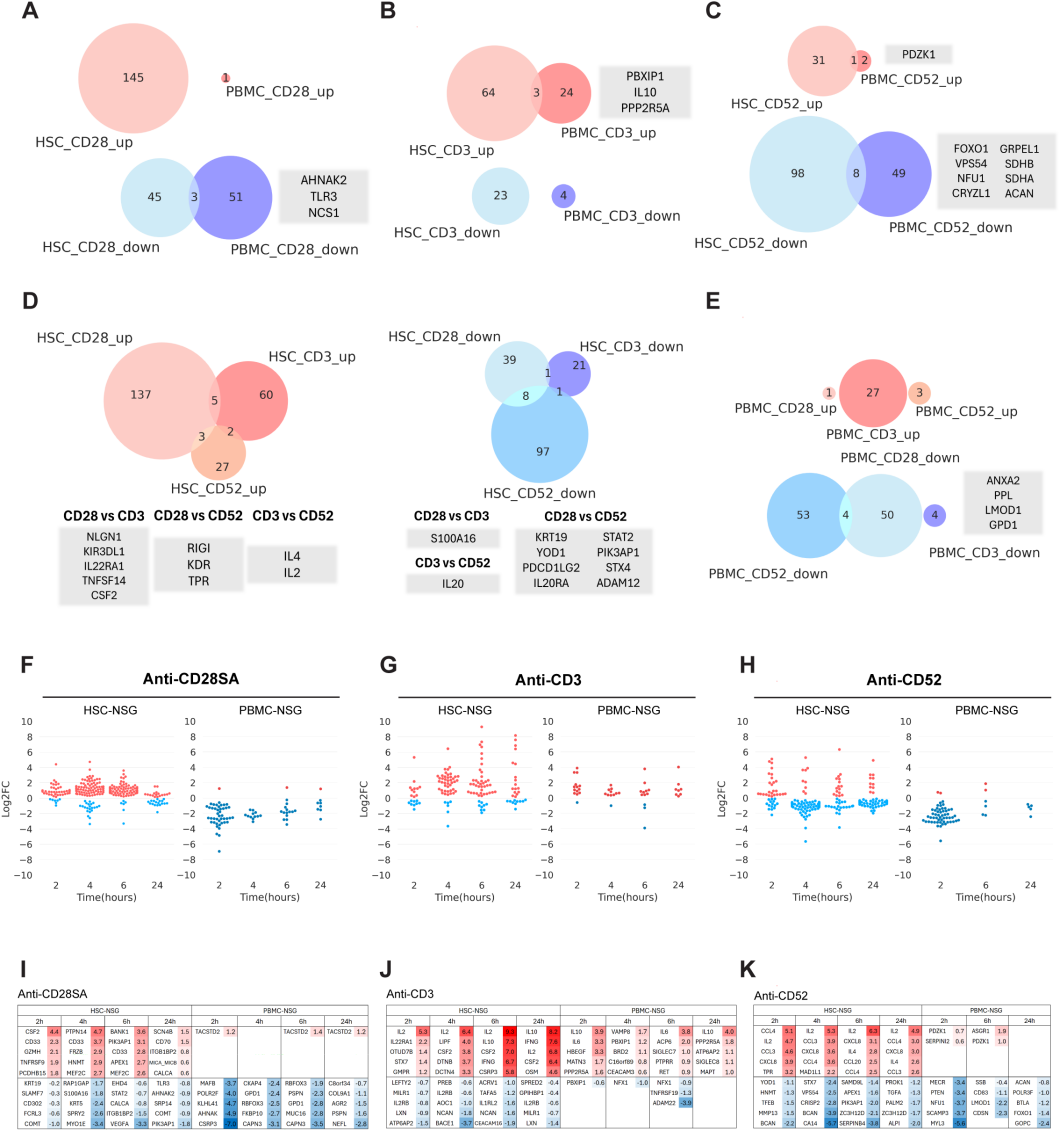
Differential secretome profiles in HSC- or PBMC- engrafted NSG mice following administration, 19/156, reference Ab panel. Plasma samples collected from engrafted NSG mice at 2,4,6 and 24 h post treatment with the reference Ab panel were selected (n=1-2) by randomisation. Plasma samples with volume of 4μl obtained from selected samples was used for Olink proximity extension assay (PEA). Timepoint-specific differentially expressed proteins (DEP), upregulated (left) or downregulated (right) were identified by comparing treatment-control pairs using linear mixed-effect regression (LMER) and *post-hoc* analysis. DEPs from all timepoints were combined and compared for Venn diagram generation. Comparative analysis of DEPs between HSC- versus PBMC-engrafted NSG mice are represented as Venn diagrams for the indicated treatment groups **(A)** anti-CD28- SA, **(B)** anti-CD3, and **(C)** anti-CD52 treatments, respectively. Overall comparison of DEPs in **(D)** HSC- or **(E)** PBMC-engrafted NSG mice is represented. **(F–H)** Swarm plot representation of quantities and log_2_ fold change (Log2FC) of DEPs at 2, 4, 6, 24h following treatment with **(F)** anti-CD28- SA, **(G)** anti-CD3, and **(H)** anti-CD52 treatment in HSC- or PBMC-engrafted NSG mice. **(I–K)** Top 5 up or down regulated DEP, corresponding to each indicated treatment, ranked by Log2FC, are tabulated.

As envisaged from the MSD assay results discussed above, the overall number of DEPs were higher in HSC-engrafted NSG mice, across all the three positive test Abs in the reference panel (**Figures 8A–C**). The molecular signatures of HSC- versus PBMC-engraftment models exhibited limited overlap with only 3 to 9 DEPs identified as a total of both upregulated and downregulated proteins. Similarly, the DEP overlap among the three test Abs were minimal, ranging from a total 0 to 11 DEPs, highlighting distinct expression profiles across the treatment-model combinations (**Figures 8D, E**). However, few cytokine DEPs upregulated in more than one condition include IL-10 (**Figure 8B**); tumour necrosis factor superfamily protein 14 (TNFSF14); CSF2, more commonly known as granulocyte macrophage colony-stimulating factor (GM-CSF); IL-4; and IL-2 (**Figure 8D**). Consistent with the number of DEPs, the fold changes of upregulated DEPs in the HSC-engraftment model were generally higher compared to the PBMC-engraftment model, while downregulation intensities were comparable. In HSC-engrafted NSG mice, anti-CD28- SA and anti-CD3 treatment led to complex patterns of upregulation, anti-CD3 and anti-CD52 induced robust increases in certain DEPs, while anti-CD52 resulted in larger number of downregulated DEPs. In the PBMC-engraftment model, anti-CD3 treatment produced prominent upregulation of several DEPs, whereas anti-CD28- SA and anti-CD52 elicited distinct and substantial downregulation signatures within the first two hours (**Figures 8F–H**).

Despite, most cytokine changes following anti-CD28- SA treatment being insignificant, (**Figures 4**, **5**), complex molecular signatures were observed particularly in the HSC-engraftment model. Upregulation of T-cell activation related or associated proteins including CSF2, granzyme H (GZMH), TNF-receptor superfamily 9 (TNFRSF9) or CD137, upon anti-CD28- SA treatment indicate T-cell engagement (**Figure 8F**, **Supplementary Figure 6A**), while increase in B-cell scaffold protein with ankyrin repeats 1 (BANK1) and phosphoinositide-3-kinase adaptor protein 1 (PIK3AP1), implied enrichment of B-cell signalling and development pathways respectively; myeloid cell lineage related proteins such as CD33/Siglec-3, myocyte enhancer factor 2C (MEF2C); protein tyrosine phosphatase 14 (PTPN14), frizzled-related protein (FRZB), histamine N-methyltransferase (HNMT) proteins were also identified in HSC-engraftment model, following anti-CD28- SA treatment (**Figures 8F, I**). Cytokine signatures following anti-CD3 treatment in the HSC-engraftment model were coincident with four of the five cytokines also found significant in MSD assays: IL-2, IL-10, TNF-α and IFN-γ (**Figures 8G, J**, **Supplementary Figure 6C**). Other upregulated soluble mediators include CSF2 and IL-4, also observed with anti-CD28- SA treatment. In contrast, anti-CD3 induced signatures in the PBMC-engraftment model identified IL-10 and IL-6, two of the four cytokines detected with the MSD assay, together with increased secretion of heparin-binding epidermal growth factor-like growth factor (HBEGF), known to be produced by monocytes, macrophages and innate lymphoid cells (ILCs). Anti-CD52-induced signature identified IL-2 as a predominant early cytokine in HSC-engrafted mice, as also detected by the MSD assay (**Figures 4**, **8H, K**). In addition, IL-4, and chemokine factors such as C-C motif ligand 3 (CCL3), also known as macrophage inflammatory protein-1-alpha (MIP-1-alpha); CCL4 or MIP-1-beta, CCL20 or MIP-3; and chemokine (C-X-C motif) ligand 8 (CXCL8) or IL-8; involved in leukocyte/monocyte/granulocyte recruitment, migration, and chemotaxis were observed to be upregulated (**Figures 8H, K**). The above upregulated cytokines and chemokines were persistent during the 24h observation window, thus could be potential markers of anti-CD52 responses. Although the overall cytokine patterns from the Olink PEA and MSD-based analysis were similar, Olink PEA failed to capture the significant increase in IL-2 observed in anti-CD28- SA treated HSC-engrafted or following anti-CD3/anti-CD52 treatment in PBMC-engrafted NSG mice. Similarly, the IL-6 increase following anti-CD3-treatment in PBMC-engraftment model and TNF-α increase after anti-CD3 and anti-CD52 treatment in PBMC and HSC-engraftment model respectively could not be detected, potentially attributable to the small sample size and donor variability.

Significant proportion of downregulated DEPs were unrelated to immune cell functions. Based on the single-cell RNA expression database from Human Protein Atlas, they were either identified as ubiquitous such as desmoyokin (AHNAK), POLR2f- encoded DNA-directed RNA polymerases I, II, and III subunit (RPABC2), Golgi-associated PDZ and coiled-coil motif-containing protein (GOPC), mitochondrial trans-2-enoyl-CoA reductase (MECR), secretory carrier membrane protein 3 (SCAMP3) etc; or typically enhanced in specific tissues such as cysteine and glycine-rich protein 3 (CSRP3) in cardiomyocytes; neurofilament light polypeptide (NEFL), RNA-binding FOX protein family 3 (RBFOX3), neurocan (NCAN), a disintegrin and metalloproteinase domain 22 (ADAM22) etc. in neurons; calpain 3 (CAPN3), carbonic anhydrase 14 (CA14), brevican (BCAN) etc. in glial cells and persephin (PSPN), cysteine-rich secretory protein 2 (CRISP2) in late spermatids or male reproductive tract. The relevance of these proteins within the immune context remains unclear. Immune-related downregulated DEPs that are typically highly expressed in monocytes (V-maf musculoaponeurotic fibrosarcoma oncogene homolog B (MAFB), phosphoinositide 3-kinase adapter protein 1 (PIK3AP1), ATPase H^+^-transporting lysosomal accessory protein 2 (ATP6AP2), phosphatase and tensin homolog (PTEN)); granulocytes (latexin (LXN), serpin family B member 4 (SERPINB4)), or related to T and B lymphocytes (zinc finger CCCH domain-containing protein 12D (ZC3H12D), forkhead box protein O1 (FOXO1)), respectively were detected across experimental conditions, the interpretation of which will require further investigation. Overall, these results reveal both shared and unique pattern and kinetics, thereby suggesting treatment-specific protein signatures that may also be specific to a particular engraftment model (**Figure 8J**, **Supplementary Figures 6C, D**).

Mapping the DEPs to UniProt for their subcellular localisation revealed that, while secreted factors were key contributors, additional molecular signatures primarily originating from the plasma membrane (PM) and nucleus (**Supplementary Figure 7**) were observed. Upregulated DEPs, in anti-CD28- SA and anti-CD3 treated HSC-engrafted mice and anti-CD3 treated PBMC-engrafted mice showed a predominant PM localisation, while upregulated DEPs in anti-CD52-treated HSC-engrafted mice were dominated by secreted factors. Similarly, downregulated DEPs, were observed to be primarily PM localised. Overall, the varying subcellular DEP distributions among treatment groups suggest differential cellular functions, warranting further studies (**Supplementary Figures 7A–E**).

### 3.5 *In vitro* cytokine release response to the reference Ab panel 19/156, in donor PBMCs utilised for NSG engraftment

**Figure 9:**
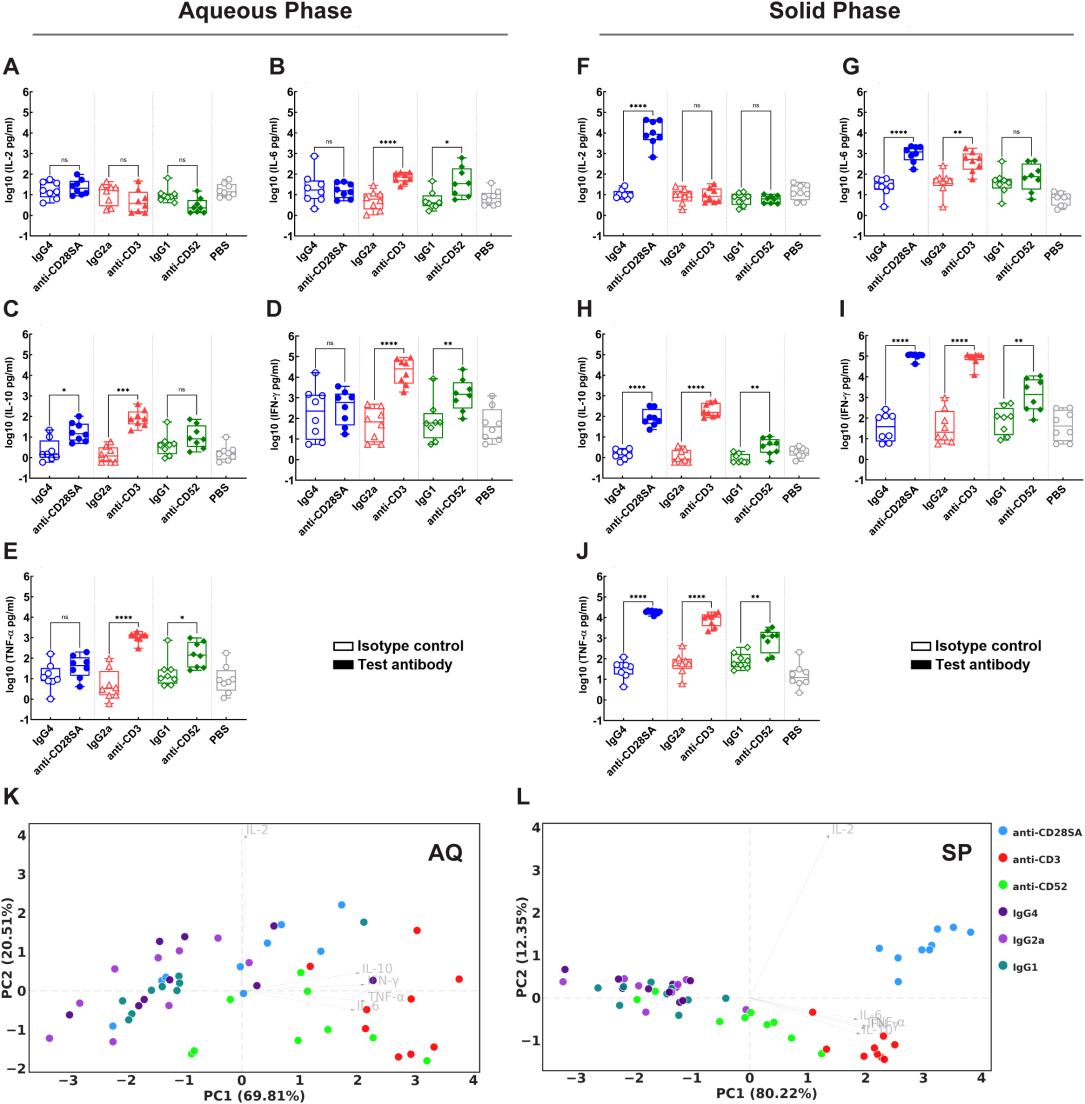
Analysis of *in vitro* cytokine release potential of donor PBMCs used for engraftment experiments. PBMCs from donors D1, D2, D4, D9, D10, D15, D17 or D20, were treated *in vitro* either with positive control test (filled symbols): **(A–J)** anti-CD28- SA (blue), anti-CD3 (red) or anti-CD52 (green); or respective isotype control Ab (hollow symbols): IgG4 (blue), IgG2a (red) or IgG1 (green). Ab was presented either in **(A–E)** aqueous phase (AQ) or **(F–J)** solid phase (SP). Quantification of cytokine levels for IL-2, IL-4, IL-6, IL-10, IFN-γ and TNF-α in culture supernatants 48h post Ab treatment was performed by MSD multiplex assays following manufacturer’s instructions. The plots represent log_10_ transformed values for level of each indicated cytokine in picograms/ml. Statistical analysis was performed using GraphPad Prism version 10.4.1 as detailed in Materials and Methods. **(K, L)** PCA biplots representing overviews of cytokine data from PBMC cultures presented with Ab in **(K)** AQ or **(L)** SP for 48 hours. The coordinates of arrowheads in the PCA biplots are 4-fold scaled eigenvectors. The unscaled eigenvectors represent the correlation of PC scores to the corresponding features. p ≤ 0.05 (*), p ≤ 0.01 (**), p ≤ 0.001 (***), p ≤ 0.0001 (****), not significant (ns).

PBMCs from the same donors as those used for NSG engraftment were additionally evaluated *in vitro* for CR potential of the reference Ab panel, presented in aqueous (AQ) or solid phase (SP) (**Figure 9**, **Supplementary Table 5**).

No significant IL-2 induction was observed for any of the positive control Abs in the AQ-CRA (**Figure 9A**). Treatment with anti-CD28- SA in AQ-CRA resulted in a specific significant 6-fold induction of IL-10 (**Figure 9C**). In contrast, anti-CD3 treatment led to a significant release of IL-6, IFN-γ, and TNF-α (9-fold, 246-fold, 65-fold increase, respectively), in addition to IL-10 (62-fold increase), while anti-CD52 treatment was associated with a comparatively lower but marked increase in IL-6, IFN-γ, and TNF-α (14-fold, 4-fold, 3-fold increase, respectively), with a lower IL-10 induction (2-fold) (**Figures 9A–E**). Significant IL-2 release was observed exclusively following anti-CD28- SA treatment in SP-CRA, in addition to all the other tested cytokines IL-6, IL-10, IFN-γ, and TNF-α. Notably this was the sole condition where *in vitro* IL-2 induction (1552-fold) was observed. The pattern of increased levels of IL-6, IL-10, IFN-γ, and TNF-α observed following anti-CD3 treatment was similar for both AQ (with fold increases of 9, 62, 246, and 65, respectively) and SP-CRA (with fold increases of 9, 176, 497, and 96, respectively). The overall fold increases were greater in SP-CRA for most cytokines. In contrast to the AQ-CRA, anti-CD52 treatment in SP-CRA, induced a significant (~5-fold) IL-10 induction. Anti-CD52 treatment significantly boosted IFN-γ and TNF-α levels in SP-CRA (22-fold and 12-fold) compared to the observed lower levels in AQ-CRA. IL-6 showed minimal 2-fold induction in SP-CRA, but was notably higher with AQ-CRA (14-fold). Notably, SP-CRA exhibited a graded pattern of highly significant cytokine production, for all five assessed cytokines (IL-2: 1552-fold, IL-6: 38-fold, IL-10: 76-fold, IFN-γ: 1261-fold, and TNF-α: 425-fold) following anti-CD28- SA treatment. In comparison, four cytokines (IL-6: 9-fold, IL-10: 176-fold, IFN-γ: 497-fold, and TNF-α: 22-fold) were induced by anti-CD3, showing lower fold increases for all except IL-10; whereas only three cytokines (IL-10: 5-fold, IFN-γ: 22-fold, and TNF-α: 12-fold), with a comparatively lower fold increase were elicited by anti-CD52. In contrast, while anti-CD28- SA only induced IL-10 (6-fold) in AQ-CRA, anti-CD3 stimulation was observed to induce four of the five assessed cytokines (IL-6: 9-fold, IL-10: 62-fold, IFN-γ: 246-fold, and TNF-α: 65-fold), while anti CD52 induced three (IL-6: 14-fold, IFN-γ: 4-fold, and TNF-α: 3-fold). These findings show that cytokine induction patterns vary with Ab presentation to responder cells and are closely tied to the functional properties of the test Ab.

Dimensionality reduction of *in vitro* cytokine profiles by PCA reveals high association of all cytokines except for IL-2 in both AQ- and SP-CRA datasets, as indicated by the similar eigenvectors across cytokines (**Figures 9K, L**). PC1 explained 69.8 to 80.2% of total variance in both *in vitro* datasets, driven almost equally by IL-6, IL-10, IFN-γ, and TNF-α, while IL-2 was the sole contributor to PC2 scores especially in AQ-CRA where progression in PC2 mainly reflect changes in IL-2 levels. Donor variability was generally high in AQ-CRA as indicated by the loose clusters except for IgG1, whereas effect of test Abs was more significant and consistent in SP-CRA as indicated by the tight and discrete clusters. Anti-CD28- SA triggered strong IL-2 release alongside other cytokines in SP-CRA but had limited effect in AQ-CRA as demonstrated by the weak PC scores and treatment-control overlap, consistent with the MSD analysis. Both anti-CD3 and anti-CD52 failed to trigger IL-2 release but had relatively comparable performance pattern across both the *in vitro* CRA formats.

## 4 Discussion

An important aspect of clinical translation involves developing and validating biological assays predictive of clinical outcomes by assessing both risk and efficacy. Our results present a pilot study reporting the first *in vivo* assessment of the reference Ab panel, 19/156, employing two distinct approaches for human cell reconstitution in NSG- derived humanised mouse model systems: HSC- and PBMC- engraftment. The reference Ab panel comprises of three immune-modulating positive control mAbs and the respective isotype matched negative controls. The positive control Abs are recognised for eliciting stratified responses corresponding to severe, moderate, and mild CR in clinical settings and have earlier been evaluated for performance as positive reference standards for *in vitro* CRA in an international collaborative study (8). Theralizumab, anti-CD28-SA, was discontinued from development in 2006 after severe CRS and subsequent chronic organ failure were identified during its first-in-human study (4). Similarly, muromonab, a CD3-targeting mAb, initially approved for treatment of acute rejection in transplant patients, was withdrawn from use in 2010 due to side effects including CRS (20, 21). Alemtuzumab, a CD52-directed cytolytic Ab used for relapsing multiple sclerosis and B-cell chronic lymphocytic leukaemia (CLL), carries significant safety warnings including the risk of CRS (22).

### 4.1 Broad immune cell reconstitution in HSC- versus T-cell dominant engraftment pattern PBMC-reconstituted mice

Consistent with published work, results presented in the current study show that HSC engraftment facilitated progressive reconstitution of various hematopoietic lineages, including T-cells, B-cells, along with NK- and NK-T cells (**Figures 1**, **2**). In contrast, the PBMC-model reconstitution was predominated by T-cell lineage cells including CD4^+^, CD8^+^ and NK-T-cells with minimal B-cell and NK-cell reconstitution (**Figure 3**) (23, 24). The study design incorporated multiple donors to account for variation in donor responses. Though there was inter-donor variability in the absolute cell counts for both the HSC- and PBMC-engraftment models, the kinetics and pattern of engraftment were similar across donors (**Figures 1**–**3**). Donor variability observed in matched donor CRA performed *in vitro* was considerably lower in comparison to *in vivo* data from PBMC- engrafted mice (**Figures 5**, **9**). Administration of anti-CD28- SA did not impact the cellular composition in either *in vivo* model, while T-cell directed, anti-CD3 lead to discernible T-cell depletion in both models (**Figures 6**, **7**). Treatment with anti-CD52 recapitulated the cytolytic nature of the Ab, resulting in depletion of all immune cell subsets in HSC-engrafted mice (**Figure 6**) and selective T-cell depletion in PBMC-engrafted mice, which already exhibit a predominant T-cell lineage reconstitution (**Figure 7**). In the absence of robust supportive signals for myeloid engraftment, both HSC and PBMC- engrafted NSG were not expected to harbour myeloid lineages proficiently. Yet, interestingly, significant non-lymphocyte populations were found in both models (**Figures 6**, **7J–L**). Complete depletion of the non-lymphocyte population in HSC-engraftment model, following anti-CD52 treatment is suggestive of considerable CD52 expression in the population, whereas only limited depletion was observed in the PBMC-engraftment model. Responses to anti-CD52 are reported to vary based on the surface CD52 expression (25) and differences in constitution of these immune cell subsets may reflect the differential cytokine pattern observed in response to anti-CD52 treatment *in vivo*. Although CD52 and CD16 expression levels on donor immune cell subsets could not be investigated within the present study, due to the inability to source cells from the same cohort; donor-specific CD52 expression could contribute to the observed differential cytokine response patterns for IL-10, IL-6, IFN-γ and TNF-α observed *in vitro*, the latter three cytokines also reported in first-dose CRS post CD52-targeting, Campath-1 administration (14). Similarly, cellular depletion may influence the induction of cytokines such as IL-2 since autocrine amplification loop could be impacted due to depletion of cytokine-producing cells following treatment.

Interestingly, analysis of post-treatment cellular profiles in HSC-engrafted mice revealed that immune cell counts across all subsets and treatment groups, measured from cardiac bleed samples collected at the point of termination, were lower (4-6-fold lower human CD45^+^) than pre-treatment values obtained from tail bleed samples obtained at week 22 (**Figures 2**, **6**). These results indicate that the site of sample collection is important, and that comparisons should be conducted using samples obtained with similar sampling conditions and/or assayed alongside isotype-matched, negative controls to ensure accurate assessment of treatment effects (26). In summary, human HSC engraftment facilitates a broader differentiation into various human immune cell types over the evaluated five-month period, whereas PBMC engraftment, established rapidly, within approximately one week, primarily results in a T-cell-dominant engraftment profile.

### 4.2 Incorporation of reference panel 19/156 allows identification of differences and similarities in cytokine profiles *in vivo* and by *in vitro* assays

Cytokine patterns following treatment with the reference Ab panel focussing on IL-2, IL-6, IL-10, IFN-γ and TNF-α known to mediate proinflammatory cascade, were next evaluated.

Analysis of CR patterns *in vivo* and *in vitro* demonstrated that anti-CD28- SA treatment resulted in early and transient IL-2 induction exclusively in the HSC-engraftment model (**Figure 4A**),mirroring the sole SP-CRA condition in which IL-2 was detected *in vitro* (**Figures 9A, F**), carefully replicating human clinical response. *In vivo* IL-2 induction was observed in response to both anti-CD3 and anti-CD52 in HSC and PBMC-engrafted mice (**Figures 4F**, **5F**), with the induction kinetics reminiscent of the clinical response, where IL-2 serum levels were found to peak by 4h in muromonab treated renal allograft patients (12). Thus *in vivo* NSG models appear to better recapitulate IL-2 induction for the reference panel Abs in comparison to *in vitro* CRA.

*In vivo* IL-6 and IL-10 were detected only in response to anti-CD3, also well captured by *in vitro* assays. However, for CD28- SA and anti- CD52, IL-6 and IL-10 induction was identified only with *in vitro* assays, with minimal/insignificant induction *in vivo*. Consistent with previous research, our results demonstrate that only SP-presentation of anti-CD28- SA induces IFN-γ *in vitro* (**Figures 9F, I**), which serves as an effective predictive marker for TGN1412-like CRS (27). Interestingly, while anti-CD3 was the only treatment that led to IFN-γ induction in HSC-engrafted mice, minimal induction was observed in PBMC-engrafted NSG mice to any of the positive control Abs. However, both *in vitro* formats effectively captured the IFN-γ response to anti-CD3. In contrast, anti-CD52 treatment did not result in significant IFN-γ release in either *in vivo* model while significant IFN-γ release was observed with both AQ and SP presentation (**Figures 4**, **5**). Previous studies have identified that distinct cellular sources, activated CD4^+^ T cells, CD4^+^ and CD8^+^ T-cells or NK cells contribute to IFN-γ induction following treatment with TGN1412, muromonab and Campath-1, respectively (28). A major limitation of the PBMC model, also encountered in the present study, is the quick onset of xenogeneic graft-versus-host disease (GvHD), which additionally reduces the time duration available for assessing candidate biologics. Despite our assessment of various donor PBMCs, all PBMC-engrafted mice in the present study exhibited elevated IFN-γ levels post engraftment regardless of treatment (**Figure 5**), indicating the possible onset of xenogeneic GvHD in PBMC- engrafted mice (29–31). Consequently, although treatment with positive control Abs lead to increments in IFN-γ response, the results were not statistically significant (**Figure 5**). Notably, no changes in clinical signs such as body weight changes, piloerection, or hunching, were observed in the engrafted mice, possibly due to the lower Ab concentrations employed in our study (~0.8 mg/kg) compared to other reports (~2 mg/kg). While a direct comparison of absolute cytokine levels with published studies is therefore challenging, the cytokine kinetics observed in our study are consistent with previous findings, confirming distinct patterns between HSC- (32–34) and PBMC-engrafted mice (33, 35, 36). Importantly, *in vitro* CRA with the same PBMCs did not result in elevated background IFN-γ responses to the isotype control or PBS, indicating that CR is driven in the mouse host post-engraftment and that the donor cells are not pre-activated while isolation (**Figure 9**). Notably, a similar donor cell immune reactivity driving GvHD and consequently leading to higher IFN-γ levels was not observed in HSC-engrafted NSG mice, where significant IFN-γ levels was induced in response to anti-CD3 treatment. However, it is not possible to directly attribute the observed elevated IFN-γ solely to GvHD, as assessment of murine inflammatory markers and histopathology could not be conducted within the scope of the present study. This study used NSG mice expressing mouse MHCI/II, but for longer term studies and avoiding the onset of acute xenogeneic GvHD, advanced models such as mice deficient in murine MHCI/II may be preferable (33, 36, 37). MHCI/II deficient mice exhibit differences in the patterns and timing of cytokine induction and are demonstrated to better replicate clinical responses to the anti-CD28- SA and anti-CD3. In summary, as noted above *in vivo* models were limited in the ability to recapitulate the IFN-γ response for anti-CD28- SA and anti-CD52, and while the SP-CRA leads to IFN- γ induction with all the reference panel Abs, AQ-CRA results in significant IFN-γ production only with anti-CD3 and anti-CD52, in line with the ability of the SP-format in capturing the requirement for receptor cross-linking in the clinical response to anti-CD28- SA-like therapeutics.

TNF-α is a significant cytokine contributing to inflammatory CRS observed in clinical responses associated with both TGN1412 and Campath-1H (38, 39). The statistical significance in TNF-α induction observed with *in vitro* SP-CRA aligns with the severity patterns documented in clinical trials, specifically anti-CD28- SA > anti-CD3 > anti-CD52 (**Figure 9**). Notably, both *in vivo* models and AQ-presentation of anti-CD28- SA exhibited minimal TNF-α levels, attributable to the inability to replicate receptor cross-linking, required for a response (27). These findings identify that the *in vivo* NSG model may have potential limitations regarding the ability to support a sufficiently functional myeloid compartment expressing CD80/86, essential for cross-linking and/or immunological synapse formation (40). Additionally, since CD4^+^ effector memory T-cells, recognised as critical mediators of pro-inflammatory responses to anti-CD28- SA (41–44), show a predominant mucosal distribution; the inability of the NSG models to effectively mirror the mucosal reconstitution may further explain why neither *in vivo* model could replicate the TNF-α response to anti-CD28- SA (43, 44). It has been demonstrated that boosting myeloid and dendritic cell populations via human Fms-like tyrosine kinase 3- ligand (Flt3L) treatment amplified CRS features, highlighting the role of these cell types in CRS development (32, 45, 46). Improved models such as the M-CSF^h/h^ IL-3/GM-CSF^h/h^ SIRPA^h/m^ TPO^h/h^ *Rag2^−/−^ Il2rg^−/−^* (MISTRG) mice (47), human *IL-15* knock-in SIRPA *Rag2^−/−^ Il2rg^−/−^* (SRG) mouse strain (48), supporting efficient myeloid and mucosal reconstitution could help overcome these limitations of the NSG mouse model, and be further evaluated for therapeutics such as anti CD28- SA, mediating adverse responses via specific immunological pathways. Further evaluation of the reference Ab panel, in improved humanised mouse models that promote NK-, myeloid cell and mucosal reconstitution following PBMC engraftment, would broaden the applicability of PBMC models for studying non-T-cell mediated mechanisms. Both SP- and AQ-format show similar TNF- α pattern for anti-CD3, although absolute quantities/levels were lower for the AQ- presentation. Interestingly, anti-CD3 produced a significant and sustained TNF-α induction in both *in vivo* and *in vitro* models. Anti-CD52 elicited a transient, early TNF-α response in HSC-engrafted mice- an effect not observed in the PBMC-engrafted NSG mice. The pattern of *in vitro* TNF-α induction in response to anti-CD52 was similar to anti-CD3 though the mean levels were comparatively lower. Notably, the pattern sustained kinetics of anti-CD3-induced TNF-α as compared to the early and transient TNF-α response elicited by anti-CD52 appears to exhibit an overall trend like the human clinical response though these responses are still temporally distinct from those observed clinically. Therefore, the *in vitro* and *in vivo* models reveal unique as well as overlapping cytokine patterns, depending on the characteristics of the test antibody.

### 4.3 Secretome analysis will provide useful insights on identification of novel *in vivo* immunotoxicological signatures

The exploratory Olink PEA analysis revealed distinct signatures showing minimal overlap in secretome profiles across the various experimental conditions, highlighting the unique functional signature for each model and positive control Abs in the reference panel. Despite the small sample size and observed donor variability, significant immune response patterns in response to the test Abs in HSC- engrafted NSG mice and to a lesser extent, in anti-CD3 treated PBMC-NSG mice were observed. Notably, GM-CSF and IL-4 both associated with CRS in COVID-19 (45, 46) and CAR-T cell toxicity (49, 50), were significantly elevated in anti-CD28- SA or anti-CD3 treated HSC-engrafted NSG mice (**Figures 8I, J**), underscoring the clinical relevance of these models in CRS risk evaluation, although similar increases were not observed in clinical trial of the two test Abs (4, 51). The cytolytic anti-CD52 Ab, bearing the IgG1-Fc region, triggered release of several chemokines linked to myeloid activation, including CCL3, CCL4, CCL20, and CXCL8 in HSC-engrafted NSG mice (**Figure 8K**), supporting the utility of the HSC-engraftment model in evaluating Fc-dependent myeloid activation. Mapping identified molecular signatures to UniProt annotations indicated associations with diverse subcellular localisation. The interpretation of circulating proteome in engraftment models comprised of heterogenous human cell types is challenging and can be further complicated by active cell depletion specific to a biologic, and renal and hepatic clearances *in vivo*. Consistent with previous studies (52), a substantial proportion of identified signatures in the experimental samples were mapped as non-secretory (**Supplementary Figure 7**). These proteins maybe presumed to enter the extracellular space following ectodomain cleavage, via ectosome/exosome release, apoptosis or cell lysis (53–55). The Olink panel did not include exosome (tetraspanins) and cell damage markers (such as lactate dehydrogenase (LDH), high mobility group box 1 protein (HMGB1), histone etc.), limiting the ability to mark the cellular activities discussed above. However, the analysis identifying increased PM localisation among upregulated non-secretory DEPs, alongside low apoptotic markers (such as caspases) and/or modest increase in cytosolic and nuclear proteins, suggest that ectodomain cleavage and ectosome could be prominent cellular activities in HSC-engrafted NSG mice treated with anti-CD28- SA or anti-CD3 (**Supplementary Figures 7A, B**). The downregulation of membrane proteins in anti-CD52-treated HSC-engrafted mice, points to reduced ectodomain shedding and ectosome release, likely linked to early cell depletion. Ectosomes are known for their immunoregulatory role and response to stimulation (55). The sustained elevation of the myeloid marker, CD33, following anti-CD28- SA treatment in HSC-engrafted NSG mice, could suggest active ectosome activity linked to stimulated myeloid cells (**Figure 9I**). Overall, extending the exploratory proteome analysis, presented in the current study, may help identify novel immunotoxicity biomarkers and offer new insights in immunotoxicology. Further research with larger sample size, advanced bioinformatics and employing orthogonal methods to validate the findings will therefore be valuable.

### 4.4 Study limitations

The present study has certain limitations. Firstly, the cohort size for individual donors in both the HSC- and PBMC- engraftment models is small and future studies with expanded cohorts would strengthen and improve confidence in the performance evaluation of the CR panel *in vivo*. Additionally, sex-based differences in experimental animals and/or donors could not be captured within the present study. Further collaborative studies for evaluation of the reference panel in improved and advanced *in vivo* model systems will provide better understanding of limitations and strengths of specific models. Second, comprehensive analysis of immune cell subsets, with extended kinetic analysis following treatment was constrained due to limitations in the volume and frequency of the tail bleed samples permitted under the HO licence specifications for the current study. Future studies using multiparameter spectral flowcytometric and comprehensive kinetic analysis for both human and mouse cytokines/cell markers in engrafted mice will provide useful insights. Third, as also indicated above, extending the proteomic analysis across a broader sample set would further substantiate the findings of the proof-of-concept data presented in the current manuscript. Fourthly, the present study evaluated a low dose of the Abs due to severity limits specified in the HO licence and future dose escalation studies could provide useful data for better alignment of observed CRS profiles to clinically relevant doses.

## 5 Conclusions

Parallel evaluation of the reference panel in both *in vitro* and *in vivo* settings as presented in our study, enables a comprehensive analysis of the utility of the reference Ab panel, 19/156. Further interlaboratory studies will support the inclusion of reference Ab panels such as 19/156, to improve confidence for harmonisation of preclinical toxicological assessment. Notwithstanding the above limitations, results presented in this manuscript identify interesting parallelism and distinct features in response to three clinically diverse immunomodulatory Abs within the reference Ab panel, when evaluated *in vitro* and *in vivo*. Our study identifies that the *in vitro* SP-CRA, supports the generation of a broader cytokine response to anti-CD28- SA reminiscent of the clinical pattern, while the *in vivo* models are limited to capturing the IL-2 characteristic, only observed in HSC-engrafted mice. In contrast, anti-CD3 effects are broader and well represented within both *in vitro* models with AQ and SP presentations and *in vivo* models, except for IL-2 which is only detected *in vivo* with overlapping kinetics in HSC- and PBMC-engrafted mice. Notably TNF-α was the sole cytokine observed *in vivo* with HSC-engraftment coincident with the *in vitro* cytokine profile and clinical response to Campath-1. *In vivo* engraftment additionally elicits an IL-2 response, with similar kinetics in both HSC and PBMC-engrafted mice following anti-CD52 administration, while the *in vitro* assays are unable to capture IL-2 induction. *In vitro* assays could at times be limited due to their inability to capture the pharmacodynamic features and cellular interactions that occur *in vivo* due to the closed vessel format of these assays. The hazard identification potential of human cell engraftment-based *in vivo* humanised mouse models, on the other hand, is heavily reliant on the features and mechanistics of human immune cell reconstitution within the murine background. Based on the results in the current pilot study, we conclude that the predictive value of *in vivo* versus *in vitro* models and the clinical relevance of each experimental model is greatly dictated by the mechanism of action for the individual test therapeutic. For each of the positive control Abs, a particular model could sometimes capture the predominant clinical phenotype or at times only certain aspects. Therefore, a combination of *in vitro* and *in vivo* assays is required to provide a comprehensive insight for assessing CRS risk in preclinical safety evaluation of biologics. Incorporation of reference standards such as the reference Ab panel, 19/156, within the experimental design, enhances the comprehensive understanding of the nuances of design, mode of action and functional characterisation of a candidate therapeutic in addition to guiding the careful choice of a suitable model for preclinical testing. Such reference Ab panels will also facilitate the assessment of the translational relevance of improved next-generation models that overcome limitations of the NSG models and could more accurately mirror the clinical immunological sequence by allowing improved myeloid and mucosal reconstitution as well as incorporate strategies for mitigation of GvHD within the experimental timeframe. In the context of safety assessment for biologics, a false positive is perhaps less of an issue compared to a false negative that may in turn lead to eventualities like the TGN1412 disaster. In summary, incorporating reference materials and standards, such as the reference Ab panel, 19/156, outlined in this study, will improve understanding of the limitations and strengths inherent to each preclinical experimental model. Inclusion of the reference materials will be especially valuable for multi-site preclinical data comparisons, serving as a useful benchmark and supporting refinement and reduction in animal experimentation. The approach will inform the careful selection and design of appropriate models and support harmonisation of preclinical safety assessments, ultimately facilitating patient access to safer medicines.

## Data Availability

The raw data supporting the conclusions of this article will be made available by the authors, without undue reservation.
