# Supplementary material for: Evaluation of a reference antibody panel for prediction of cytokine release in humanised mouse models in vivo
Source: Front Immunol. 2026 Mar 20;17:1736130. doi: 10.3389/fimmu.2026.1736130 (PMC13047165; doi:10.3389/fimmu.2026.1736130)
Supplement: Supplementary file 1 [file SupplementaryFile1.zip › Supplementary Tables 1-5.pdf]

Supplementary Table 1

A

Pre-treatment	IgG4			CD28-SA			IgG2a			CD3			IgG1			CD52		
**Cell subset count/ µl**	**Mean**	**SEM**	**n**	**Mean**	**SEM**	**n**	**Mean**	**SEM**	**n**	**Mean**	**SEM**	**n**	**Mean**	**SEM**	**n**	**Mean**	**SEM**	**n**
**hu CD45$^+$ cells**	461.51	80.37	4	465.65	134.92	5	465.37	181.09	4	409.07	93.30	5	699.73	128.69	5	409.26	69.81	5
**CD20$^+$ B cells**	239.29	74.84	4	264.56	88.73	5	286.71	120.31	4	236.22	67.08	5	292.39	104.27	5	247.41	51.93	5
**CD3$^+$ T cells**	117.00	22.45	4	89.56	25.86	5	70.48	23.40	4	68.85	9.99	5	258.61	123.36	5	58.74	18.74	5
**CD4$^+$ T-cells**	52.03	11.04	4	42.25	11.52	5	34.34	12.05	4	31.60	4.18	5	135.36	76.80	5	27.08	8.43	5
**CD8$^+$ T-cells**	25.53	3.97	4	17.19	5.82	5	15.02	5.16	4	13.25	2.21	5	35.19	13.85	5	12.43	4.66	5
**CD56$^+$ NK cells**	73.95	43.17	4	51.61	16.93	5	64.02	32.66	4	35.63	11.05	5	57.17	18.49	5	33.78	5.36	5
**CD56$^+$CD3$^+$ NK-T-cells**	10.91	2.07	4	11.44	3.53	5	6.90	1.67	4	6.44	2.16	5	75.60	58.59	5	5.62	2.04	5

B

Post-treatment	IgG4			CD28-SA			IgG2a			CD3			IgG1			CD52		
**Cell subset count/ µl**	**Mean**	**SEM**	**n**	**Mean**	**SEM**	**n**	**Mean**	**SEM**	**n**	**Mean**	**SEM**	**n**	**Mean**	**SEM**	**n**	**Mean**	**SEM**	**n**
**hu CD45$^+$ cells**	79.26	14.07	4	104.86	67.57	5	93.0	61.34	4	122.4	76.12	5	196.15	93.76	5	4.54	1.53	5
**CD20$^+$ B cells**	45.76	11.86	4	84.37	57.46	5	60.2	42.94	4	100.0	66.20	5	52.16	15.70	5	3.19	1.41	5
**CD3$^+$ T cells**	16.8	7.68	4	2.90	0.64	5	12.0	6.40	4	0.4	0.35	5	53.37	31.36	5	0.10	0.06	5
**CD4$^+$ T-cells**	8.1	3.71	4	0.9	0.41	5	6.1	3.23	4	0.3	0.29	5	30.17	20.13	5	0.07	0.03	5
**CD8$^+$ T-cells**	4.5	1.89	4	1.2	0.30	5	3.0	1.77	4	0.01	0.01	5	10.00	4.57	5	0.02	0.02	5
**CD56$^+$ NK cells**	14.84	4.86	4	27.76	20.57	5	14.3	6.38	4	30.9	16.66	5	21.74	9.32	5	1.78	0.39	5
**CD56$^+$CD3$^+$ NK-T-cells**	5.80	1.70	4	2.34	1.31	5	5.9	2.34	4	0.6	0.36	5	25.05	15.26	5	0.17	0.09	5

Supplementary Table 2

A

Pre-treatment	IgG4			CD28-SA			IgG2a			CD3			IgG1			CD52		
Cell subset count/ µl	Mean	SEM	n	Mean	SEM	n	Mean	SEM	n	Mean	SEM	n	Mean	SEM	n	Mean	SEM	n
hu CD45$^+$ cells	16.05	6.07	6	10.98	2.82	6	9.41	2.41	6	11.04	3.25	6	13.84	4.32	6	12.50	3.97	6
CD20$^+$ B cells	0.51	0.13	6	0.28	0.07	6	0.16	0.06	6	0.21	0.07	6	0.34	0.18	6	0.33	0.12	6
CD3$^+$ T cells	8.76	3.43	6	5.77	1.30	6	5.27	1.11	6	6.19	1.68	6	7.65	2.32	6	6.00	1.24	6
CD4$^+$ T-cells	3.35	1.21	6	2.11	0.36	6	2.05	0.44	6	2.41	0.58	6	2.82	0.81	6	1.98	0.44	6
CD8$^+$ T-cells	1.90	0.65	6	1.34	0.31	6	1.20	0.32	6	1.42	0.46	6	1.87	0.66	6	1.35	0.22	6
CD56$^+$ NK cells	0.26	0.11	6	0.20	0.05	6	0.25	0.11	6	0.21	0.08	6	0.18	0.11	6	0.18	0.06	6
CD56$^+$CD3$^+$ NK-T-cells	0.92	0.38	6	0.51	0.15	6	0.41	0.09	6	0.51	0.18	6	0.62	0.19	6	2.42	1.92	6

B

Post-treatment	IgG4			CD28-SA			IgG2a			CD3			IgG1			CD52		
Cell subset count/ µl	Mean	SEM	n	Mean	SEM	n	Mean	SEM	n	Mean	SEM	n	Mean	SEM	n	Mean	SEM	n
hu CD45$^+$ cells	11.04	3.92	6	9.23	4.86	6	17.04	12.72	6	1.94	1.03	6	19.31	9.09	6	3.60	1.79	6
CD20$^+$ B cells	0.03	0.02	6	0.03	0.01	6	0.02	0.01	6	0.01	0.01	6	0.01	0.01	6	0.00	0.00	6
CD3$^+$ T cells	8.58	3.14	6	7.45	4.11	6	12.69	9.65	6	0.90	0.58	6	15.41	7.16	6	2.67	1.35	6
CD4$^+$ T-cells	4.12	1.62	6	2.53	1.51	6	6.14	4.98	6	0.05	0.03	6	6.22	3.28	6	0.53	0.32	6
CD8$^+$ T-cells	1.59	0.68	6	1.47	0.89	6	2.60	1.98	6	0.08	0.07	6	2.82	1.37	6	0.32	0.22	6
CD56$^+$ NK cells	0.10	0.03	6	0.08	0.03	6	0.09	0.03	6	0.13	0.08	6	0.10	0.03	6	0.06	0.03	6
CD56$^+$CD3$^+$ NK-T-cells	1.71	0.69	6	2.46	1.56	6	3.07	2.53	6	0.65	0.40	6	2.60	1.14	6	0.91	0.41	6

Supplementary Table 3

	Time (h)	IgG4			CD28-SA			IgG2a			CD3			IgG1			CD52		
		Mean	SEM	n	Mean	SEM	n	Mean	SEM	n	Mean	SEM	n	Mean	SEM	n	Mean	SEM	n
IL-2	**2**	3.6	0.0	4.0	**35.7**	14.5	5.0	3.6	0.0	4.0	102.3	42.7	5.0	3.6	0.0	4.0	112.6	65.6	5.0
	**4**	3.6	0.0	4.0	25.6	8.4	5.0	3.6	0.0	4.0	130.6	47.1	5.0	3.6	0.0	4.0	**134.7**	87.9	5.0
	**6**	3.6	0.0	4.0	10.3	2.8	5.0	3.6	0.0	4.0	118.3	24.6	5.0	3.6	0.0	4.0	105.7	58.2	5.0
	**24**	3.6	0.0	4.0	3.8	0.3	5.0	3.6	0.0	4.0	**154.5**	45.6	5.0	3.6	0.0	4.0	4.1	0.4	5.0
IL-6	**2**	2.5	0.0	4.0	4.0	1.0	5.0	2.5	0.0	4.0	4.9	1.4	5.0	2.5	0.0	4.0	4.6	1.3	5.0
	**4**	2.5	0.0	4.0	3.0	0.3	5.0	2.5	0.0	4.0	13.1	6.5	5.0	2.5	0.0	4.0	**11.1**	5.5	5.0
	**6**	2.5	0.0	4.0	2.6	0.1	5.0	2.5	0.0	4.0	7.4	3.0	5.0	2.5	0.0	4.0	9.4	4.7	5.0
	**24**	2.5	0.0	4.0	2.5	0.0	5.0	2.5	0.0	4.0	**20.5**	8.5	5.0	2.5	0.0	4.0	3.4	0.9	5.0
IL-10	**2**	1.2	0.0	4.0	3.4	1.2	5.0	1.4	0.2	4.0	13.2	6.9	5.0	1.5	0.3	4.0	7.5	3.4	5.0
	**4**	1.2	0.0	4.0	**4.1**	2.2	5.0	1.5	0.3	4.0	40.0	23.5	5.0	1.7	0.5	4.0	12.6	6.4	5.0
	**6**	1.2	0.0	4.0	3.2	1.3	5.0	1.4	0.2	4.0	49.2	28.7	5.0	1.7	0.5	4.0	**20.8**	13.3	5.0
	**24**	1.2	0.0	4.0	1.3	0.1	5.0	1.4	0.2	4.0	**166.7**	47.1	5.0	1.4	0.2	4.0	3.3	2.0	5.0
IFN-γ	**2**	15.3	5.6	4.0	163.5	68.5	5.0	15.1	5.3	4.0	342.7	218.7	5.0	44.2	17.5	4.0	873.7	563.3	5.0
	**4**	15.2	4.9	4.0	404.6	231.3	5.0	14.7	5.5	4.0	917.8	615.8	5.0	43.7	17.4	4.0	1446.9	840.6	5.0
	**6**	16.4	5.6	4.0	**452.6**	233.5	5.0	13.8	4.1	4.0	1179.5	761.1	5.0	46.3	19.0	4.0	**1800.6**	1141.7	5.0
	**24**	16.3	6.2	4.0	175.5	87.7	5.0	12.3	3.1	4.0	**2749.9**	878.5	5.0	49.7	21.9	4.0	637.1	493.4	5.0
TNF-α	**2**	2.8	0.0	4.0	**14.7**	4.9	5.0	2.8	0.0	4.0	36.2	21.7	5.0	3.5	0.5	4.0	**47.5**	23.2	5.0
	**4**	2.8	0.0	4.0	8.6	2.3	5.0	3.1	0.4	4.0	**46.9**	30.0	5.0	3.6	0.7	4.0	35.9	19.7	5.0
	**6**	2.8	0.0	4.0	6.5	1.7	5.0	2.8	0.0	4.0	31.6	15.2	5.0	3.5	0.5	4.0	28.4	16.0	5.0
	**24**	2.8	0.0	4.0	2.9	0.1	5.0	2.8	0.0	4.0	20.9	4.8	5.0	3.5	0.5	4.0	4.5	1.2	5.0

Supplementary Table 4

	Time (h)	IgG4			CD28-SA			IgG2a			CD3			IgG1			CD52		
		Mean	SEM	n	Mean	SEM	n	Mean	SEM	n	Mean	SEM	n	Mean	SEM	n	Mean	SEM	n
IL-2	**2**	5.8	1.6	6.0	**13.8**	4.8	6.0	4.6	1.1	6.0	158.0	42.2	6.0	4.5	1.1	6.0	94.9	49.0	6.0
	**4**	4.7	1.8	6.0	**13.8**	4.3	6.0	7.2	2.2	6.0	561.7	171.1	6.0	4.8	1.3	6.0	**114.5**	56.0	6.0
	**6**	5.1	1.6	6.0	9.6	3.4	6.0	**24.1**	9.4	6.0	**830.4**	403.7	6.0	6.1	1.4	6.0	75.2	43.6	6.0
	**24**	6.7	3.0	6.0	3.4	1.1	6.0	8.1	2.6	6.0	57.2	17.4	6.0	8.8	2.9	6.0	3.5	0.9	6.0
IL-6	**2**	3.1	1.1	6.0	**4.6**	1.4	6.0	2.4	0.8	6.0	12.5	3.0	6.0	2.0	0.6	6.0	**8.2**	3.4	6.0
	**4**	3.4	1.0	6.0	**4.6**	1.7	6.0	3.8	0.8	6.0	21.5	5.1	6.0	2.9	0.8	6.0	**8.2**	2.8	6.0
	**6**	2.3	0.6	6.0	3.9	1.4	6.0	**5.3**	2.1	6.0	**22.7**	6.9	6.0	3.1	0.8	6.0	6.6	2.7	6.0
	**24**	**4.3**	2.5	6.0	1.7	0.5	6.0	3.3	1.5	6.0	5.5	2.1	6.0	**3.2**	1.6	6.0	2.0	0.7	6.0
IL-10	**2**	4.5	2.4	6.0	**18.4**	10.9	6.0	3.8	1.6	6.0	88.0	39.1	6.0	3.9	1.6	6.0	**41.9**	29.7	6.0
	**4**	3.4	1.9	6.0	11.7	6.1	6.0	9.3	3.9	6.0	93.6	36.4	6.0	5.5	2.0	6.0	29.4	18.8	6.0
	**6**	5.1	2.9	6.0	13.4	6.3	6.0	**33.6**	17.2	6.0	103.6	35.3	6.0	9.5	3.8	6.0	25.7	16.1	6.0
	**24**	**14.5**	9.9	6.0	3.8	1.5	6.0	11.1	4.9	6.0	**106.5**	38.3	6.0	**13.2**	6.5	6.0	3.5	0.9	6.0
IFN-γ	**2**	3454.7	1798.4	6.0	5444.2	3077.6	6.0	3218.0	1229.0	6.0	11425.4	3546.9	6.0	2445.0	1044.5	6.0	8340.0	4377.2	6.0
	**4**	1887.0	1134.9	6.0	**6594.7**	3601.2	6.0	3759.7	1496.2	6.0	18104.3	3892.4	6.0	2799.4	1175.8	6.0	**11081.0**	5080.7	6.0
	**6**	1872.8	1063.3	6.0	6202.5	3042.3	6.0	6119.0	1937.2	6.0	**19198.8**	4826.2	6.0	3265.8	1172.6	6.0	10773.8	5099.9	6.0
	**24**	**7021.7**	4429.2	6.0	2728.9	1270.9	6.0	**7158.1**	3665.4	6.0	18560.2	4503.5	6.0	**7820.5**	4221.0	6.0	4019.8	2412.6	6.0
TNF-α	**2**	4.6	2.7	6.0	**7.9**	3.3	6.0	2.5	0.9	6.0	77.2	28.0	6.0	2.7	1.1	6.0	**48.7**	28.4	6.0
	**4**	2.0	1.2	6.0	6.0	2.6	6.0	5.6	2.8	6.0	**144.9**	55.9	6.0	3.1	1.5	6.0	22.2	11.2	6.0
	**6**	2.9	1.5	6.0	6.1	2.3	6.0	**10.5**	5.2	6.0	130.7	82.8	6.0	4.0	1.5	6.0	11.4	5.5	6.0
	**24**	**7.1**	4.5	6.0	3.2	1.4	6.0	6.6	3.4	6.0	14.5	7.3	6.0	6.1	3.3	6.0	2.6	1.6	6.0

Supplementary Table 5

A

	PBMC-AQ	IgG4	CD28-SA	IgG2a	CD3	IgG1	CD52	PBS
IL-2	**Mean**	**23.3**	**32.3**	**17.2**	**10.4**	**15.2**	**4.2**	**21.4**
	Donor1	4.0	97.5	1.8	17.9	7.8	15.1	7.3
	Donor10	16.1	15.8	24.5	7.0	8.9	2.3	7.4
	Donor2	12.1	10.2	15.1	4.8	7.4	1.4	15.2
	Donor4	14.1	53.2	5.5	46.0	5.7	6.5	12.8
	Donor9	6.4	8.8	2.2	3.0	4.1	1.5	10.6
	Donor20	36.9	12.1	22.8	1.6	11.7	1.4	25.8
	Donor15	41.8	34.0	43.3	1.4	10.9	3.5	35.9
	Donor17	55.1	26.7	22.1	1.4	65.4	2.4	56.2
IL-6	**Mean**	**110.9**	**21.2**	**8.7**	**77.9**	**9.7**	**132.3**	**10.4**
	Donor1	2.1	31.8	1.1	25.8	2.9	36.2	3.0
	Donor10	748.5	11.0	5.2	91.4	4.2	33.1	5.3
	Donor2	57.0	5.1	3.1	42.0	2.8	5.8	3.0
	Donor4	21.3	13.6	1.6	53.4	1.6	11.9	3.6
	Donor9	13.4	43.1	10.8	58.3	11.9	8.2	14.0
	Donor20	6.0	5.8	7.3	123.0	4.0	620.1	7.9
	Donor15	6.6	17.9	12.1	109.9	4.6	227.8	8.7
	Donor17	32.5	41.4	28.4	119.7	45.8	115.2	37.7
IL-10	**Mean**	**5.0**	**29.5**	**2.0**	**123.0**	**9.7**	**20.6**	**2.5**
	Donor1	0.6	102.5	0.6	21.3	4.7	18.2	0.7
	Donor10	10.3	16.1	3.6	74.8	4.0	7.8	0.6
	Donor2	2.1	6.2	1.0	76.6	4.1	1.9	1.6
	Donor4	1.1	36.3	0.6	405.8	2.6	5.3	2.4
	Donor9	0.9	8.4	0.6	49.8	0.9	2.8	1.5
	Donor20	1.5	4.9	1.4	204.8	6.1	72.6	1.1
	Donor15	1.4	13.1	2.3	38.9	1.2	8.7	2.1
	Donor17	21.8	48.1	5.9	112.3	53.9	47.6	10.1
IFN-γ	**Mean**	**2491.7**	**1003.7**	**150.9**	**37077.8**	**1116.5**	**4769.6**	**234.4**
	Donor1	5.6	3600.9	5.6	1920.9	57.0	1688.7	5.6
	Donor10	16437.1	353.0	483.0	78515.6	35.3	1283.8	9.1
	Donor2	1474.5	35.1	48.6	7370.9	63.6	222.5	27.8
	Donor4	23.6	1061.3	13.0	13595.0	7.2	739.2	12.6
	Donor9	6.7	101.8	5.6	4215.2	5.6	95.7	63.3
	Donor20	78.9	17.2	94.1	89849.9	62.8	24118.1	55.8
	Donor15	639.9	1002.1	410.1	54160.3	255.4	2635.1	457.7
	Donor17	1267.6	1858.2	147.1	46994.3	8444.9	7373.3	1243.1
TNF-α	**Mean**	**32.1**	**65.9**	**18.8**	**1227.3**	**105.5**	**312.9**	**31.3**
	Donor1	1.0	198.2	0.6	307.3	27.8	128.8	1.1
	Donor10	161.4	26.7	3.6	1384.7	9.4	466.2	2.0
	Donor2	40.2	4.1	1.6	893.5	4.6	37.4	6.9
	Donor4	11.6	64.7	1.5	2010.9	10.4	25.6	5.7
	Donor9	7.4	32.4	3.0	1411.4	4.8	33.8	8.5
	Donor20	7.7	10.9	5.0	1495.5	13.3	977.1	9.4
	Donor15	9.2	57.6	39.9	1366.6	27.7	154.9	36.6
	Donor17	18.0	132.7	95.2	948.7	746.2	679.5	179.9

B

	PBMC-SP	IgG4	CD28-SA	IgG2a	CD3	IgG1	CD52	PBS
IL-2	**Mean**	**11.9**	**18401.7**	**11.2**	**12.7**	**7.3**	**6.5**	**17.2**
	Donor1	8.4	34148.0	3.9	33.7	4.5	9.2	11.0
	Donor10	9.0	43056.0	10.1	10.1	2.8	10.5	4.0
	Donor2	17.5	10335.5	10.7	15.7	8.2	8.4	12.3
	Donor4	10.0	4062.7	10.7	7.0	6.6	3.8	11.3
	Donor9	7.2	7329.6	1.9	21.5	2.0	3.6	4.2
	Donor20	6.0	662.6	8.3	4.9	12.4	3.8	26.3
	Donor15	27.1	6063.0	25.8	4.4	13.8	6.1	28.4
	Donor17	9.6	41556.6	17.9	4.2	7.9	6.5	39.9
IL-6	**Mean**	**33.2**	**1266.9**	**69.7**	**642.8**	**84.3**	**155.3**	**7.2**
	Donor1	2.6	397.6	2.6	57.4	3.7	6.0	8.3
	Donor10	26.6	2247.6	45.9	253.1	38.3	52.9	2.9
	Donor2	26.1	987.2	257.7	631.7	49.6	86.7	1.9
	Donor4	15.5	169.3	21.9	442.1	17.1	12.4	7.5
	Donor9	58.8	1446.1	62.3	620.6	31.7	64.2	12.1
	Donor20	24.5	781.8	48.8	1817.5	51.3	428.6	7.5
	Donor15	60.9	2016.1	65.6	1177.7	419.0	447.3	3.0
	Donor17	50.8	2089.4	53.1	142.7	63.5	143.9	14.4
IL-10	**Mean**	**1.6**	**123.9**	**1.4**	**255.0**	**1.0**	**4.7**	**2.1**
	Donor1	0.6	312.2	0.6	145.2	0.6	1.6	1.2
	Donor10	2.6	324.5	2.5	492.1	1.6	5.0	0.7
	Donor2	2.7	90.4	3.6	376.2	2.0	9.1	2.2
	Donor4	0.9	56.4	0.6	553.0	0.6	0.6	2.0
	Donor9	1.9	59.4	0.8	171.1	0.7	3.5	1.3
	Donor20	1.2	40.0	0.6	116.6	1.2	5.1	2.8
	Donor15	1.8	22.7	0.9	86.6	0.6	2.0	2.7
	Donor17	1.2	85.1	2.0	99.6	0.6	10.5	3.7
IFN-γ	**Mean**	**82.4**	**103964.2**	**177.9**	**88400.0**	**168.3**	**3661.1**	**112.0**
	Donor1	5.6	95945.6	5.6	12408.3	29.1	279.7	20.7
	Donor10	12.7	118860.0	34.7	117839.8	522.3	5347.9	5.6
	Donor2	122.3	113395.7	960.7	117351.9	123.8	8589.3	81.9
	Donor4	13.8	41697.3	12.3	57706.6	11.8	81.5	19.7
	Donor9	106.5	111314.1	12.3	94779.9	8.5	258.3	5.6
	Donor20	6.1	115742.6	7.0	111145.1	120.8	10939.6	314.2
	Donor15	266.0	115009.5	313.0	109415.5	422.0	613.8	303.2
	Donor17	126.5	119749.1	77.3	86552.7	108.4	3178.4	145.2
TNF-α	**Mean**	**43.9**	**18628.7**	**102.1**	**9789.3**	**113.3**	**1307.8**	**37.7**
	Donor1	4.3	16155.1	6.1	2200.6	26.6	115.8	7.4
	Donor10	41.1	21764.7	76.9	9996.0	105.6	1274.1	2.2
	Donor2	48.2	20093.3	421.0	18429.2	196.3	2323.9	8.3
	Donor4	15.9	11752.6	26.2	11060.1	49.2	93.3	22.3
	Donor9	119.8	19554.3	108.5	14863.4	35.0	1356.6	17.7
	Donor20	21.8	18819.1	56.8	12984.9	55.0	3384.0	30.2
	Donor15	51.1	19208.4	64.0	5433.9	358.5	688.8	6.6
	Donor17	48.8	21682.5	57.3	3346.5	79.9	1226.1	206.9
