# Supplementary material for: Evaluation of a reference antibody panel for prediction of cytokine release in humanised mouse models in vivo
Source: Front Immunol. 2026 Mar 20;17:1736130. doi: 10.3389/fimmu.2026.1736130 (PMC13047165; doi:10.3389/fimmu.2026.1736130)
Supplement: Supplementary file 1 [file SupplementaryFile1.zip › Supplementary Figure and Table legends.docx]

**Supplementary Figure Legends**

**Supplementary Figure 1:** **Representative FACS plots and gating strategy for cell count determination in the HSC- or PBMC- engrafted NSG mice**. Blood samples from experimental mice were surface stained for flowcytometric enumeration of human cells as described in the Materials and Methods. Samples were stained in BD Trucount tubes and red blood cells were lysed just prior to acquisition. Representative gates to identify populations based on perCP cy5.5 or forward scatter versus side scatter to determine (A) bead or (B) human cells, respectively in (A-F) HSC- engrafted or (G-L) PBMC- engrafted NSG mice are depicted. (C, I) Human lymphocytes were distinguished from host mouse cells using human and mouse CD45. (D, J) B cells (CD20^+^) and T- cells (CD3^+^); (E, K) NK cells (CD56^+^) and NK-T- cells (CD56^+^, CD3^+^) were identified on gated human CD45^+^ cells and (F, L) CD4^+^ and CD8 T^+^- cells were identified on gated human T- cells.

**Supplementary Figure 2**: **Pre-processing workflow for uniform manifold approximation and projection (UMAP) embedding**. Individual flow cytometry standard (FCS) files from HSC- or PBMC- engrafted NSG mice were labelled with sample ID and treatment condition using Keyword function in FlowJo, followed by concatenation. The beads and human CD45^+^ populations were gated as illustrated and subsequently exported. Human CD45^+^ events per 10,000 beads were calculated for each treatment condition, followed by normalisation for human CD45^+^ events per treatment condition as calculated by the DownSampling FlowJo plugin. The normalised gates from all treatment groups were consolidated into a single FCS file for further downstream analysis. Finally, UMAP embedding was performed on ungated events of the consolidated normalised FCS file based on fluorescent intensities for CD3, CD4, CD8, CD56 and CD20, using UMAP_R FlowJo plugin.

**Supplementary Figure 3**: **Gating strategies employed for generating UMAP-embedded FCS files.**  Distribution of each phenotypic marker within the embeddings were screened visually. Conventional gating approach was applied for markers with scattered distribution across UMAP clusters, which included (A) CD56 in the UMAP generated for the HSC-engrafted and (B) both CD56 and CD20 in the UMAP generated for the PBMC-engrafted NSG mice. For the remaining markers with cluster specific distributions, a direct gating approach based on marker intensities and clustering pattern was adopted to avoid over generalisation of when FlowSOM should be applied. Automatic gating algorithm ─ FlowSOM ─ was evaluated but not adopted in this analysis, as it did not improve the identification of rare populations and caused over-fragmentation of clusters.

**Supplementary Figure 4**: **Level of humanisation is dependent of number of CD34+ injected.** Six 4-weeks old NSG (NOD/SCID/IL2rg^-/-^) female mice were injected with CD34^+^ cells. Three different doses of CD34^+^ cells (n=2) were used to establish the best condition for the engraftment experiment; 0.05 x 10^6^ CD34^+^ cells/mouse (●), 0.15 x 10^6^ CD34^+^ cells/mouse (■), 0.45 x 10^6^ CD34^+^ cells/mouse (▲). Extent of humanisation of HSC- engrafted mice was assessed from tail bleed samples by flow cytometry. Human lymphocytes CD45^+^ were distinguished from host mouse cells using human and mouse CD45. Kinetics of engraftment for each HSC-dose for (A) Total human lymphocyte (hu CD45^+^); (B) B cell (CD20^+^); (C) T- cell (CD3^+^); (D) CD4^+^ (CD3^+^, CD4^+^), and (E) CD8^+^ (CD3^+^, CD4^+^) T- cells are represented.

**Supplementary Figure 5**: **Immune cell reconstitution pattern for each individual donor in CD34^+^ HSC-engrafted NSG mice.** CD34^+^ cells from individual donors were used for mouse engraftment. Mice were intravenously administered 100 ml of 0.15x10^6^ CD34^+^ cells in PBS from the indicated donors: (A) CB121211A (●), (B) CB121212A (■), (C) CB121101A (▲), (D) CB121030A (▼), (E) CB121108B (♦). Total human lymphocyte (hu CD45^+^); B cell (CD20^+^); T- cell (CD3^+^); CD4^+^ (CD3^+^, CD4^+^), and CD8^+^ (CD3^+^, CD4^+^) T- cell; NK cell (CD56^+^); (G) NK-T- cell (CD56^+^, CD3^+^) counts for the individual donors determined using BD Trucount tubes, from tail bleed samples collected at week 22 post HSC injection are represented.

**Supplementary Figure 6**: **Complete secretome profiles in HSC or PBMC-engrafted NSG mice following administration of reference Ab panel, 19/156.** Differentially expressed proteins (DEP) identified in positive versus matched isotype control groups at 2, 4, 6, 24h post treatment are listed for (A, C, E) HSC-engrafted or (B, D, F) PBMC-engrafted NSG mice, after treatment with (A, B) anti-CD28- SA; (C, D) anti-CD3; or (E, F) anti-CD52 treatment. The DEPs were ordered according to their kinetic profiles.

**Supplementary Figure 7: Predicted subcellular locations of differentially expressed proteins (DEPs) from secretome profiles.** The accession code of each DEP from the post-hoc analysis output were mapped to the UniProt database for predicted subcellular locations. Subcellular composition of DEPs from HSC-NGS or PBMC-NSG following treatment of (A) anti-CD28- SA, (B) anti-CD3, and (C) anti-CD52 were presented in conjoined bar plots showing both up and down regulations.

**Supplementary Table Legends**

**Supplementary Table 1**: **Immune cell counts in CD34^+^ HSC- engrafted NSG mice prior to and after administration of positive or isotype control Abs.** Mice engrafted with CD34^+^ HSCs from donors CB121211A, CB121212A, CB121101A, CB121030A, or CB121108B were administered either the positive control test or matched isotype control Ab. Total human lymphocyte (hu CD45^+^); B cell (CD20^+^); T- cell (CD3^+^); CD4^+^ (CD3^+^, CD4^+^), CD8^+^ (CD3^+^, CD4^+^) T- cell; NK cell (CD56^+^); (G) NK-T- cell (CD56^+^, CD3^+^) counts for each experimental group, determined using BD Trucount tubes, from blood samples collected (A) pretreatment and (B) post treatment with indicated Ab from reference panel, 19/156, are presented alongside SEM and n.

**Supplementary Table 2: Pre- and posttreatment immune cell counts in PBMC-engrafted NSG mice.** Mice engrafted with PBMCs from donors D1, D2, D4, D9, D10, D17 or D20 were administered either the positive control test or matched isotype control Ab. Total human lymphocyte (hu CD45^+^); B cell (CD20^+^); T- cell (CD3^+^); CD4^+^ (CD3^+^, CD4^+^), CD8^+^ (CD3^+^, CD4^+^) T- cell; NK cell (CD56^+^); NK-T- cell (CD56^+^, CD3^+^) counts for each experimental group, determined using BD Trucount tubes, from blood samples collected (A) pretreatment and (B) post treatment with indicated Ab from reference panel, 19/156, are presented alongside SEM and n.

**Supplementary Table 3: Cytokine concentrations in CD34^+^ HSC-engrafted NSG mice following administration of either the positive control test or the corresponding isotype control Ab.** Mice engrafted with CD34^+^ HSC were treated intravenously with either 20 μg positive control test or respective isotype control Abs. Quantification of cytokine level for IL-2; IL-6; IL-10; IFN-γ; and TNF-α; in plasma samples from HSC- engrafted mice collected at 2h, 4h, 6h and 24h post Ab administration was performed by MSD multiplex assays following manufacturer’s instructions. Cytokine levels for indicated cytokines are represented in picograms/ml. The values representing the peak levels for each cytokine are denoted in **bold** text.

**Supplementary Table 4: Cytokine levels in PBMC-engrafted NSG mice following treatment with positive or isotype control Abs.** PBMC-engrafted mice engrafted received intravenous administration of either 20 μg positive control test or corresponding isotype control Abs. Cytokine quantification for IL-2; IL-6; IL-10; IFN-γ; and TNF-α; in plasma samples from engrafted mice collected at 2h, 4h, 6h and 24h post Ab administration was performed by MSD multiplex assays following manufacturer’s instructions. Cytokine concentrations for the indicated cytokines are shown in picograms/ml. Peak values for each cytokine are indicated in **bold** text.

**Supplementary Table 5: *In vitro* cytokine release levels measured in matched donor PBMCs.** *In vitro* CRA was performed with PBMCs from donors D1, D2, D4, D9, D10, D15, D17 or D20, treated either with positive control test or respective isotype control Abs presented either in aqueous phase (AQ) or solid phase (SP). Quantification of cytokine levels for IL-2, IL-4, IL-6, IL-10, IFN-γ and TNF-α in culture supernatants 48h post Ab treatment was performed by MSD multiplex assays following manufacturer’s instructions. The plots represent log_10_ transformed values for level of each indicated cytokine in picograms/ml. Mean values of each cytokine for the respective treatment group are presented in blue text.
