# Supplementary figures and images for: Evaluation of a reference antibody panel for prediction of cytokine release in humanised mouse models in vivo
Source: Front Immunol. 2026 Mar 20;17:1736130. doi: 10.3389/fimmu.2026.1736130 (PMC13047165; doi:10.3389/fimmu.2026.1736130)

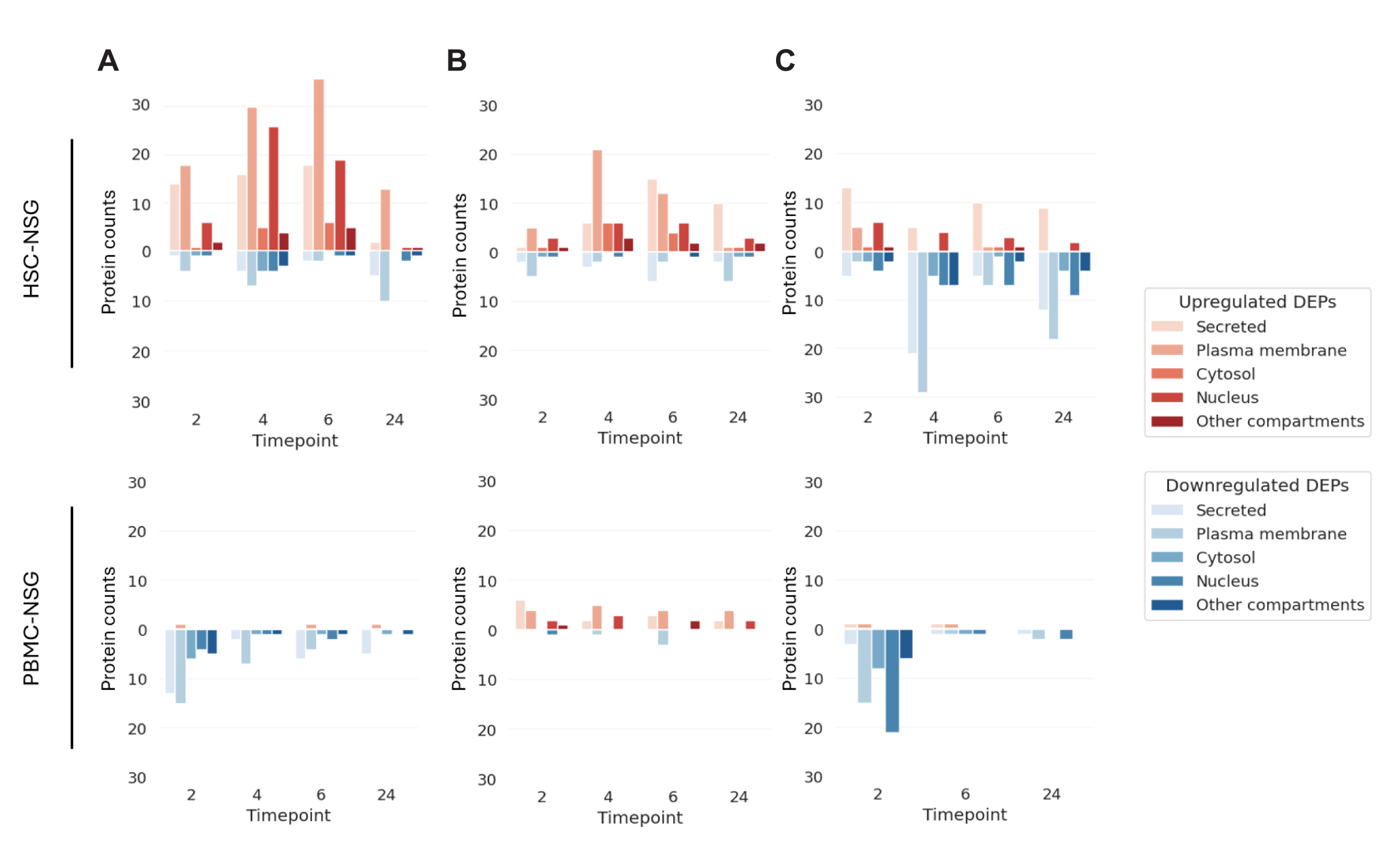

Supplement: Supplementary file 1 [file SupplementaryFile1.zip › Supplementary Figure 7.TIF]

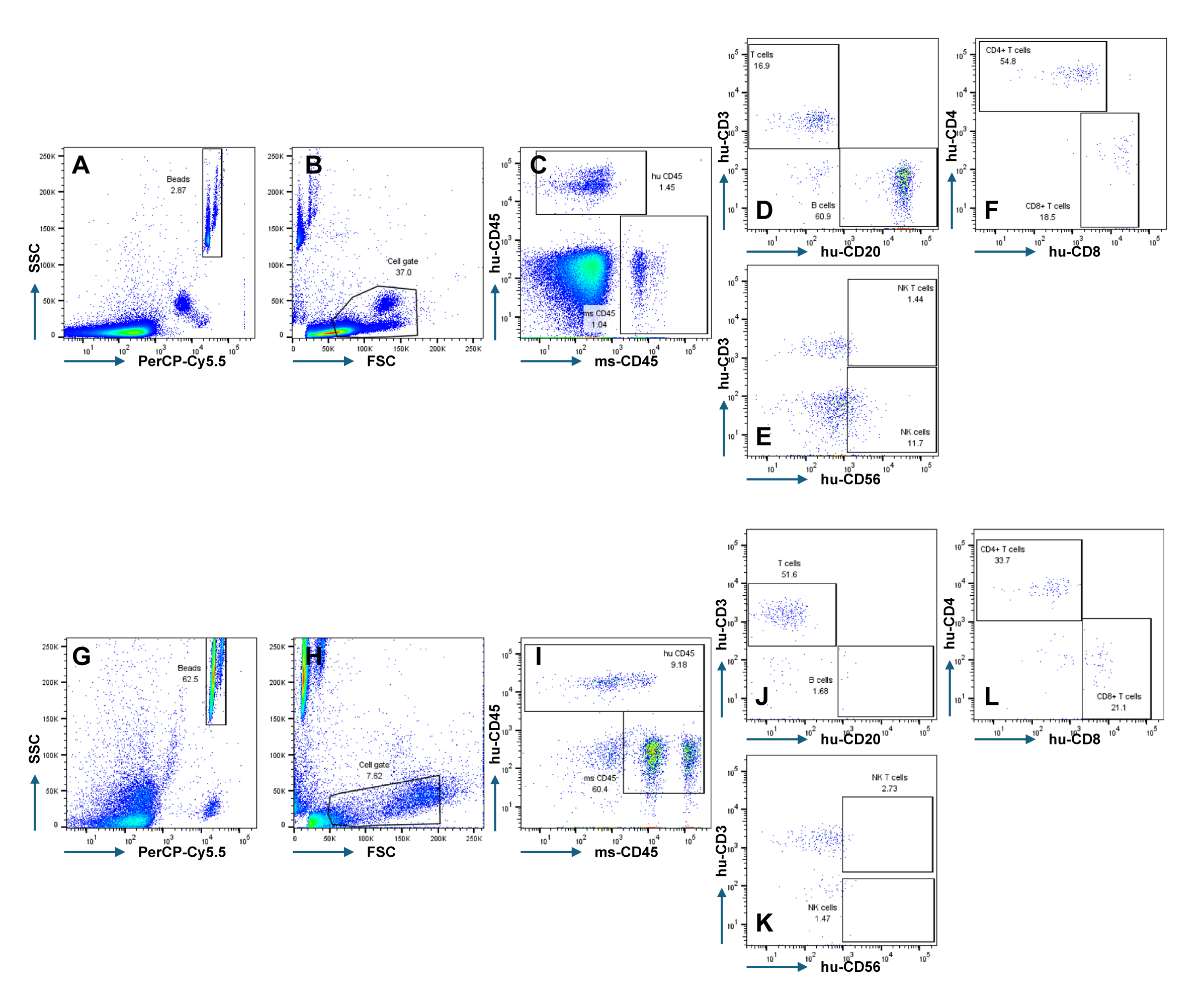

Supplement: Supplementary file 1 [file SupplementaryFile1.zip › Supplementary Figure 1.TIF]

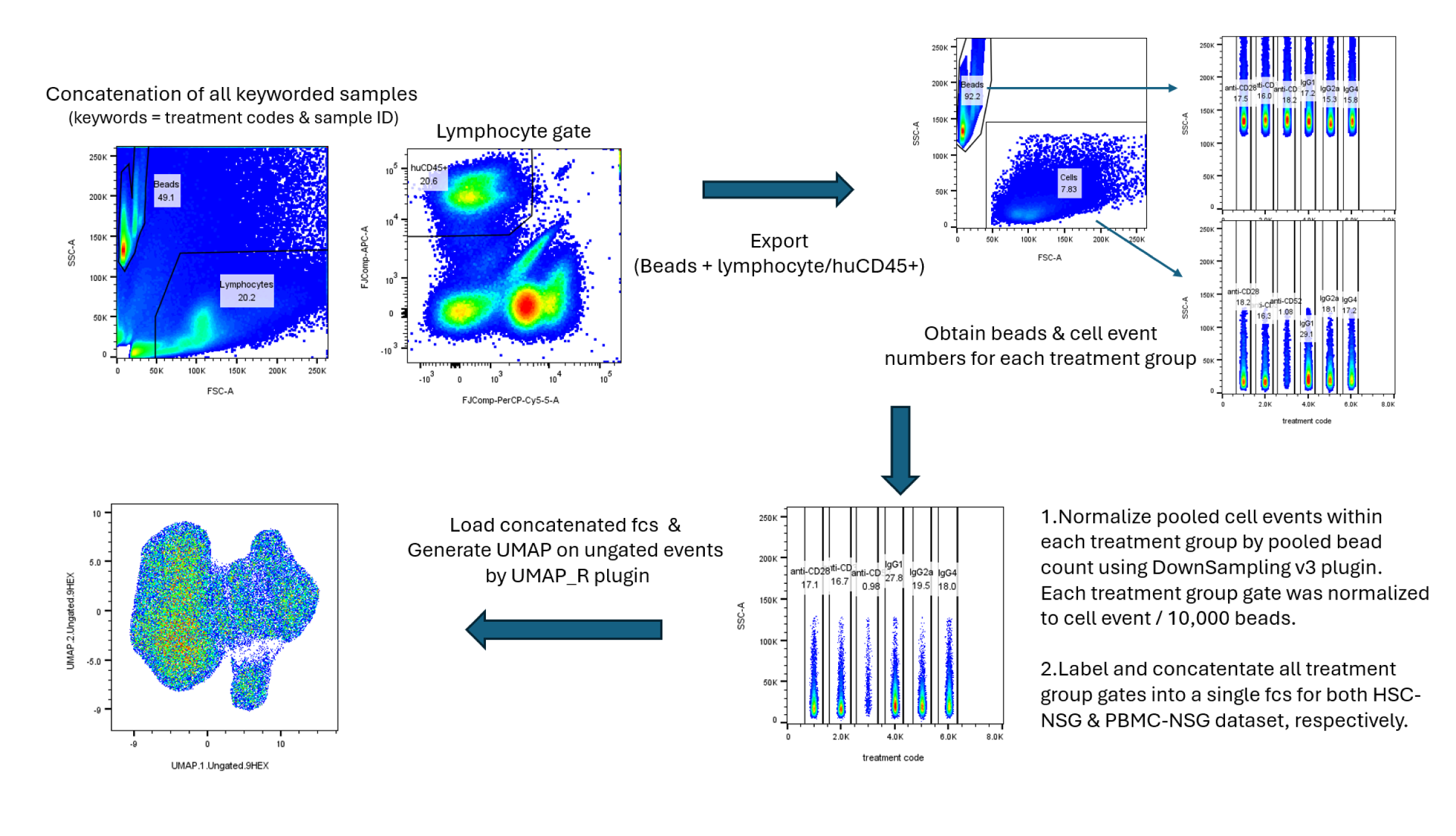

Supplement: Supplementary file 1 [file SupplementaryFile1.zip › Supplementary Figure 2.TIF]

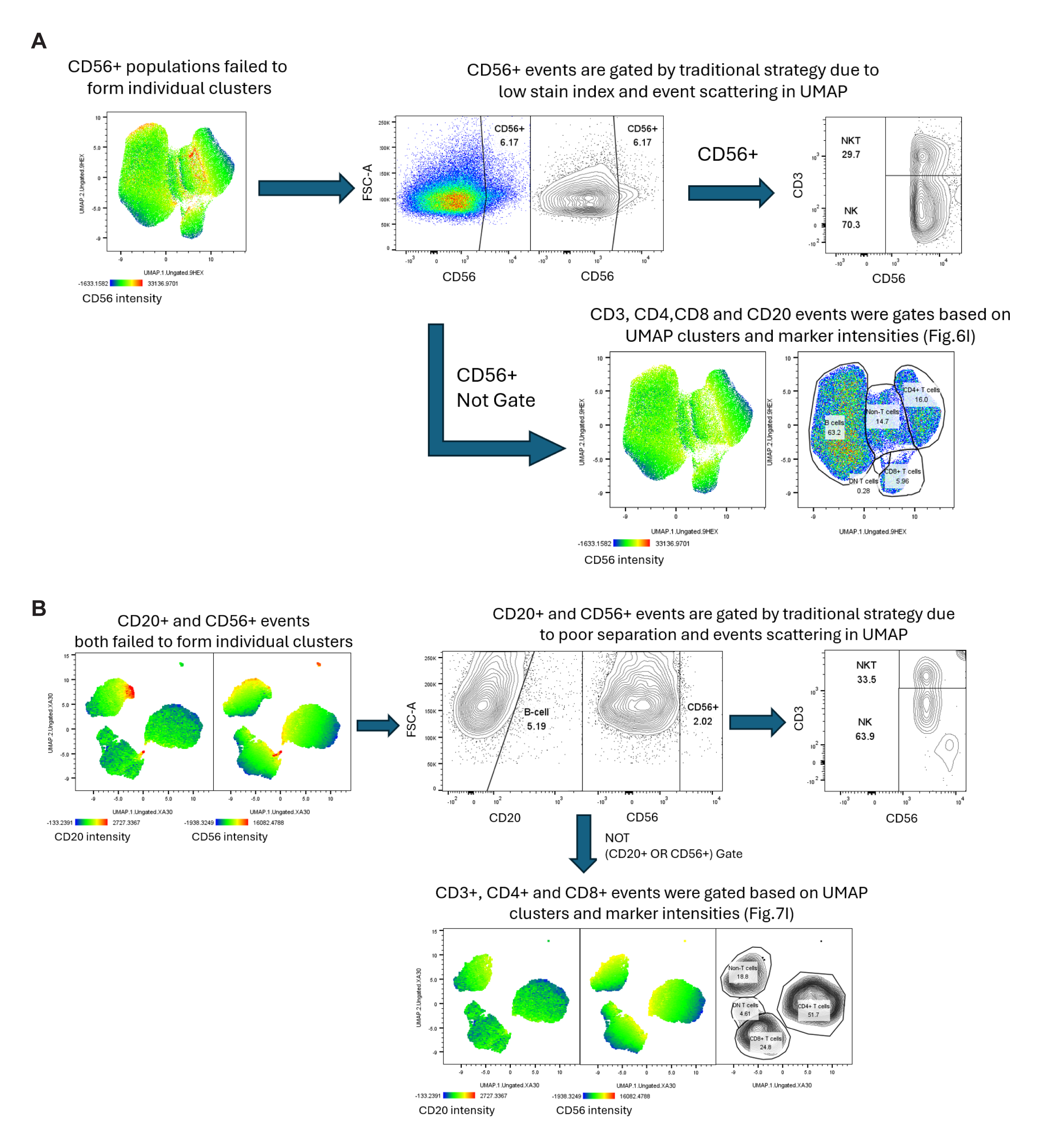

Supplement: Supplementary file 1 [file SupplementaryFile1.zip › Supplementary Figure 3.TIF]

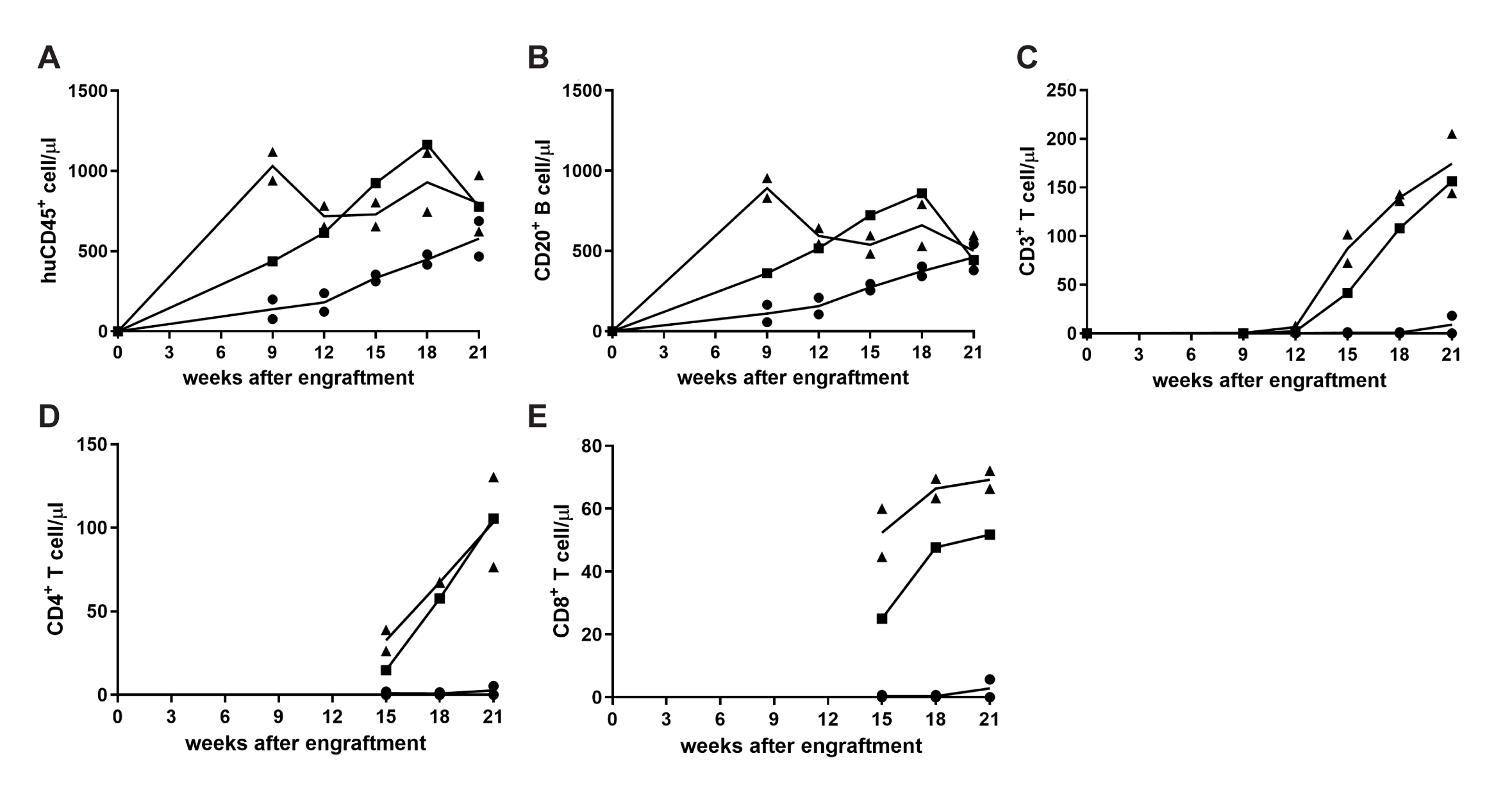

Supplement: Supplementary file 1 [file SupplementaryFile1.zip › Supplementary Figure 4.TIF]

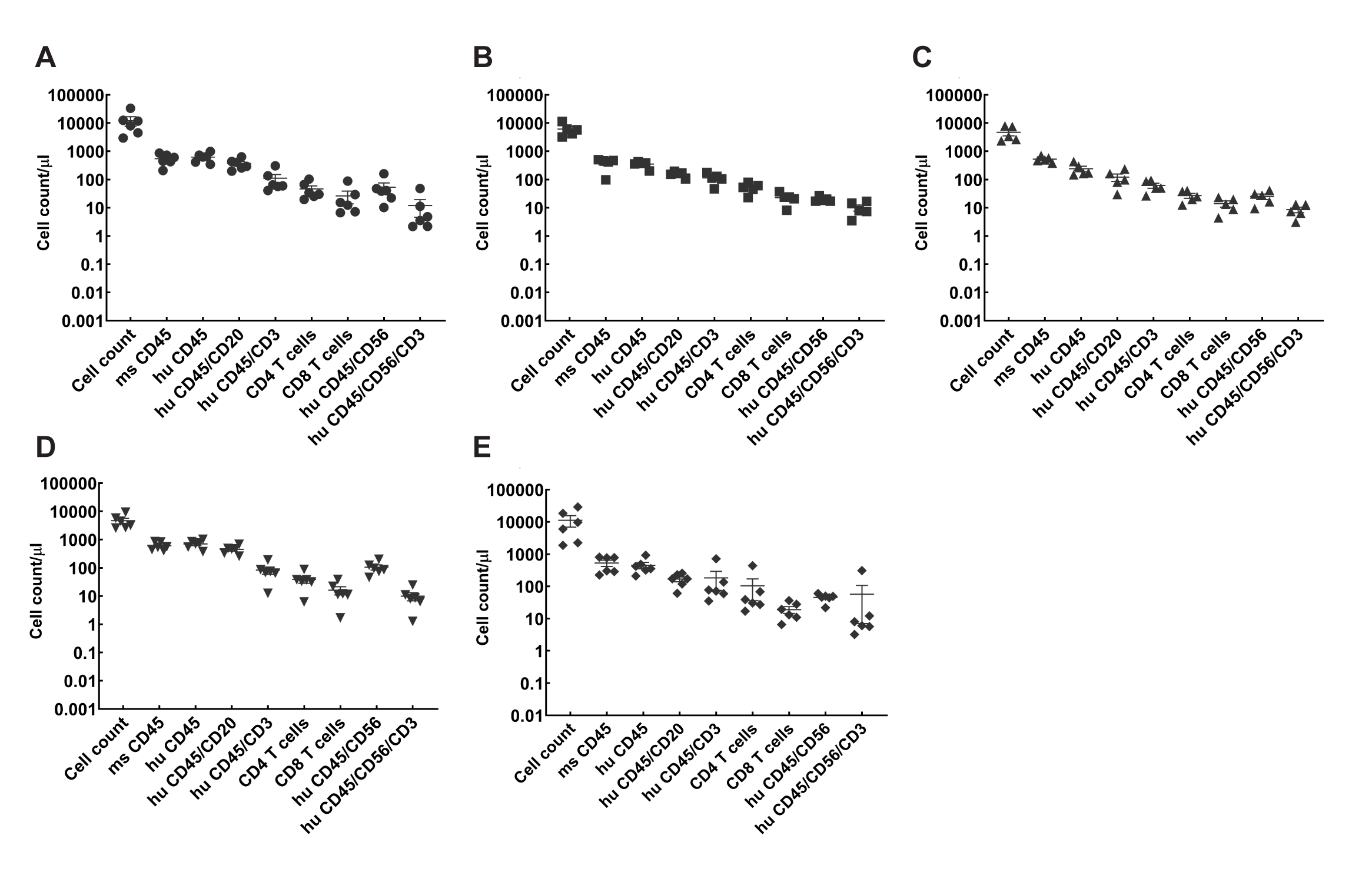

Supplement: Supplementary file 1 [file SupplementaryFile1.zip › Supplementary Figure 5.TIF]

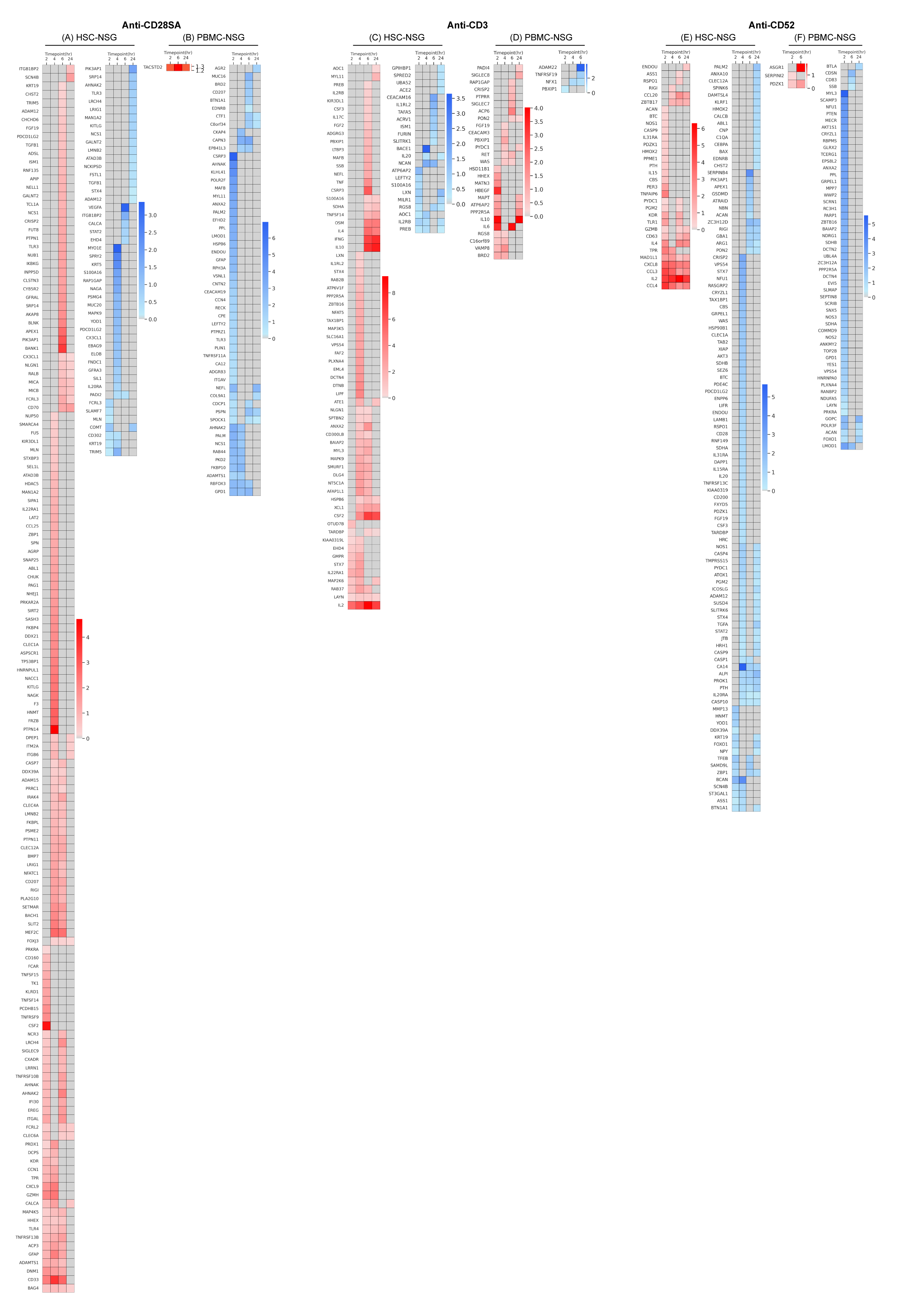

Supplement: Supplementary file 1 [file SupplementaryFile1.zip › Supplementary Figure 6.TIF]
